# A pipeline for identification of causal mutations in barley identifies Xantha-j as the chlorophyll synthase gene

**DOI:** 10.1093/plphys/kiae218

**Published:** 2024-04-17

**Authors:** David Stuart, Shakhira Zakhrabekova, Morten Egevang Jørgensen, Christoph Dockter, Mats Hansson

**Affiliations:** Department of Biology, Lund University, Sölvegatan 35B, 22362 Lund, Sweden; Department of Biology, Lund University, Sölvegatan 35B, 22362 Lund, Sweden; Carlsberg Research Laboratory, J. C. Jacobsens Gade 4, 1799 Copenhagen V, Denmark; Carlsberg Research Laboratory, J. C. Jacobsens Gade 4, 1799 Copenhagen V, Denmark; Department of Biology, Lund University, Sölvegatan 35B, 22362 Lund, Sweden

## Abstract

Thousands of barley (*Hordeum vulgare* L.) mutants have been isolated over the last century, and many are stored in gene banks across various countries. In the present work, we developed a pipeline to efficiently identify causal mutations in barley. The pipeline is also efficient for mutations located in centromeric regions. Through bulked segregant analyses using whole genome sequencing of pooled F_2_ seedlings, we mapped 2 mutations and identified a limited number of candidate genes. We applied the pipeline on F_2_ mapping populations made from *xan-j.59* (unknown mutation) and *xan-l.82* (previously known). The Xantha-j (*xan-j*) gene was identified as encoding chlorophyll synthase, which catalyzes the last step in the chlorophyll biosynthetic pathway: the addition of a phytol moiety to the propionate side chain of chlorophyllide. Key amino acid residues in the active site, including the binding sites of the isoprenoid and chlorophyllide substrates, were analyzed in an AlphaFold2-generated structural model of the barley chlorophyll synthase. Three allelic mutants, *xan-j.19*, *xan-j.59*, and *xan-j.64*, were characterized. While *xan-j.19* is a 1 base pair deletion and *xan-j.59* is a nonsense mutation, *xan-j.64* causes an S212F substitution in chlorophyll synthase. Our analyses of *xan-j.64* and treatment of growing barley with clomazone, an inhibitor of chloroplastic isoprenoid biosynthesis, suggest that binding of the isoprenoid substrate is a prerequisite for the stable maintenance of chlorophyll synthase in the plastid. We further suggest that chlorophyll synthase is a sensor for coordinating chlorophyll and isoprenoid biosynthesis.

## Teaser

A gene identification pipeline reveals the barley gene encoding chlorophyll synthase and proposes its role as an isoprenoid substrate sensor in coordinating chlorophyll and isoprenoid biosynthesis.

## Introduction

Barley (*Hordeum vulgare* L.) is one of the most economically important cereals, prompting intensive investigation into induced barley mutants since the 1920s (Lundqvist 1992). Today, barley cultivars grown worldwide contain several mutations that were induced by physical (e.g. X-ray) or chemical (e.g. sodium azide) treatment. Thousands of mutants that were produced are today stored in various gene banks (Hansson et al. 2024). These reservoirs of genetic diversity serve as a valuable asset for future plant breeding. The mutants are also of scientific interest since identifying defective genes associated with the observed mutant phenotypes provides a gateway to unraveling plant molecular processes. Traditionally, the identification of genes defective in barley mutants relied on findings from other organisms (Hansson et al. 2018). This changed with the publication of the 5.3 Gb barley reference genome (Mascher et al. 2017), coupled with advancements in genomic DNA sequencing techniques. There is now a great potential to harness barley mutants to identify and comprehend the causal genes behind observed traits. One of the more powerful gene identification techniques, which is often used for smaller genome species, is bulked segregant analysis (BSA; Michelmore et al. 1991) coupled with whole genome sequencing. Numerous specialized bioinformatics pipelines have been developed for such experiments (Schneeberger et al. 2009; Abe et al. 2012). The basic principle of this technique is to pool individuals from a mapping population based on presence or absence of the mutant phenotype. Whole genome resequencing is then performed on the “mutant” and “wild-type” pools. Genetic markers, usually SNPs, are then compared between the 2 pools. If, for example, the mutation is a single recessive allele, then markers completely linked to the mutant phenotype will be fixed for the allele from the mutant parent in the mutant pool. In the wild-type pool, the same markers will have an allele ratio of 1:2 for mutant parent to the wild-type parent alleles. On the other hand, markers that are completely unlinked will have a 1:1 allele ratio in both pools, while markers that show partial linkage will have ratios in-between. The power of this approach comes from the fact that one obtains both positional information from the allele frequency distributions to map the genomic region and whole genome sequencing data that can be used to directly identify candidate mutations. As whole genome sequencing can still be prohibitively expensive for large genome species, this technique has largely been limited to smaller genome species. In large genome species such as barley, various complexity reduction approaches have been applied to bulked segregant analysis coupled with next-generation sequencing. For example, by performing exome sequencing (Mascher et al. 2014), RNA-seq (Liu et al. 2012), or restriction site-associated DNA sequencing (RAD-seq) on the pooled samples (Xu et al. 2019). The complexity reduction techniques, although reducing the sequencing costs, have their own drawbacks. For example, exome capture may miss sequencing the causal mutation if the gene is not in the exome capture panel. Furthermore, exome capture is not readily available for all species. RNA-seq may also miss the causal mutation if the gene is not expressed in the sampled tissue, and the allele frequencies may be distorted by altered expression of genes between the mutant and wild-type individuals. RAD-seq is unlikely to provide direct sequence data of the causal mutation since only small fragments of the genome are sequenced. We applied the BSA-seq approach on 2 barley chlorophyll mutants, *xan-j.59* and *xan-l.82*. By not sequencing wild-type pools and instead using the other mutant pool as a control, cost was effectively decreased by 50% per sample. While the Xantha-l (*xan-l*) gene has previously been identified as encoding the magnesium protoporphyrin IX monomethyl ester cyclase (Rzeznicka et al. 2005), the *xan-j* gene had not been identified. Three allelic mutants are available at the *xan-j* locus: *xan-j.19*, *xan-j.59*, and *xan-j.64*. The mutants belong to the Xantha group of mutants, which are characterized by a yellow phenotype due to presence of carotenoids but absence of chlorophyll (Henningsen et al. 1993). We identified *xan-j* as chlorophyll synthase. This enzyme is responsible for attaching the phytol moiety (geranylgeranyl pyrophosphate [GGPP] or phytyl pyrophosphate [PhyPP]) to the carbon-17 propionate side chain of chlorophyllide, which is the last step of chlorophyll biosynthesis (Emery and Akhtar 1987). We describe the 3 *xan-j* mutants and the effects of the mutations. We suggest that in vivo enzyme stability is dependent on binding of GGPP/PhyPP to the enzyme active site and propose that chlorophyll synthase functions as a sensor for coordinating chlorophyll and isoprenoid biosynthesis.

## Results

### Identification of causal mutations

**Figure 1.**
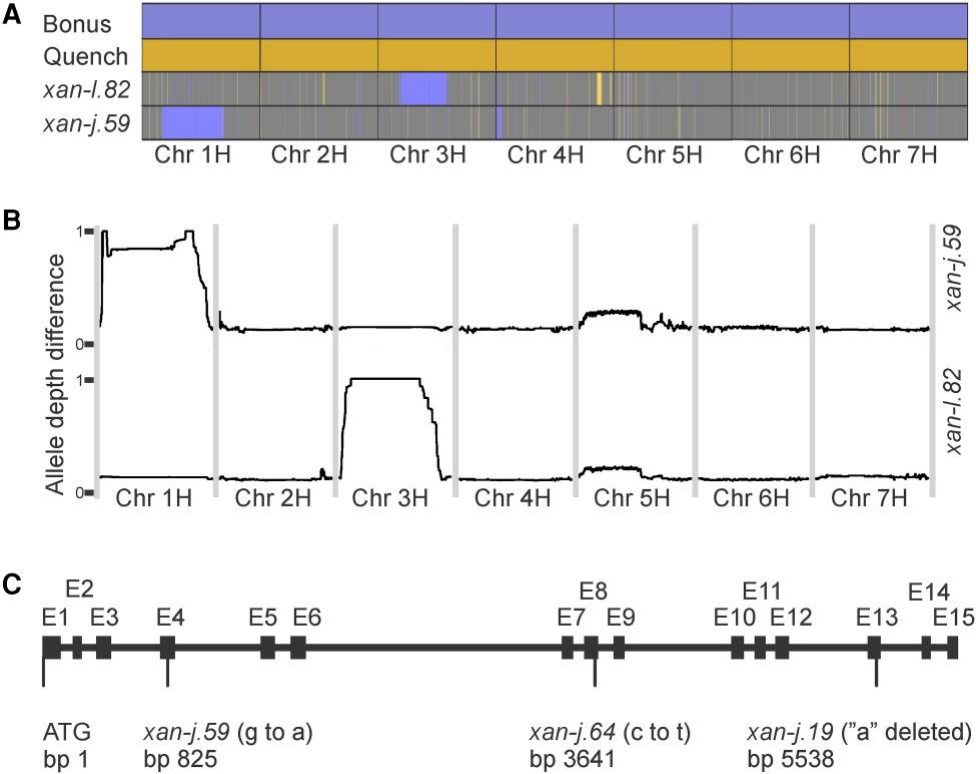
Gene mapping and gene structure. **A)** Mapping of *xan-j.59* and *xan-l.82* with phenotypic bulks from F_2_ mapping populations using a 50k SNP chip. In the figure, the SNPs are placed in consecutive order on the respective chromosome (chr) and not according to their bp position in the physical map of the barley genome. The mutant genotypes were compared to those of Bonus and Quench. Bonus is the parental cultivar of the 2 mutants, which had been crossed to cultivar Quench to generate the mapping populations. Alleles from Bonus are colored blue and alleles from Quench are colored orange. Heterozygous SNPs are gray. The large blue regions on chromosomes 3H and 1H indicate the relative position of the *xan-l.82* and *xan-j.59* mutation, respectively. **B)** Mapping of *xan-j.59* and *xan-l.82* using the allele depth difference for each bulk (the absolute value of the difference between read counts for 2 alleles divided by the sum of the read counts for the 2 alleles, giving values between 0 and 1). The *xan-j.59* mutation clearly maps to chromosome 1H with 2 small candidate regions with a median allele depth difference of 1. The *xan-l.82* mutation is known to be located in the centromere of chromosome 3H, and this can be seen by the large centromeric region with an allele depth difference of 1 on chromosome 3H for the *xan-l.82* bulk. **C)** Gene structure of *xan-j*. The gene is 6,052 bp and consists of 15 exons marked E1 to E15. Base pairs are numbered from the first bp of the ATG start codon. The deduced polypeptide is 377 amino acid residues including the transit peptide. The *xan-j.59* and *xan-j.64* mutations are single nucleotide substitutions causing an early truncation of the protein and substitution of Ser-212 to Phe, respectively. The *xan-j.19* mutation is a single base pair deletion adding a few nonnative amino acid residues followed by an early truncation.

In order to map the chromosomal location of the mutations behind the yellow Xantha phenotype, the mutants *xan-j.59* and *xan-l.82* were crossed to the cultivar Quench. Since the mutations are lethal in homozygous form, the plants used for crosses came from heterozygous stocks which segregated into green and yellow plants. While the yellow homozygous mutants soon died, the green plants, which are a mixture of heterozygous mutants and wild type, were used in the crosses. Because the green phenotypes of the heterozygous mutants and the wild-type plants are indistinguishable, the progenies had to be sorted to get an F_2_ mapping population based on heterozygous F_1_ plants. To do this, the F_1_ plants were grown to full maturity. One spike with F_2_ seeds from each F_1_ plant was planted. The appearance of yellow and green seedlings, germinating from a spike, demonstrated the heterozygous genotype of the F_1_ plant. The remaining F_2_ seeds of that plant were kept and pooled with F_2_ seeds from other heterozygous F_1_ plants to form the F_2_ mapping population. For mapping, approximately 1,000 F_2_ seeds were planted and grown at room temperature for 1 wk. The plants were placed on the floor under the lab bench to avoid direct sunlight. For each mapping population, 200 to 300 homozygous mutant F_2_ seedlings were harvested. The leaf material was pooled, and DNA was extracted to create 1 mutant bulk sample for each mapping population. These bulks were first genotyped with the barley 50k SNP chip (Bayer et al. 2017) to ensure that the 2 causal mutations were on different chromosomes so that we could use the 2 bulks as control samples for each other when performing bulk segregant analysis by whole genome sequencing. This showed a linked region on chromosome 1H for the *xan-j.59* F_2_ bulk and a linked region on chromosome 3H for the *xan-l.82* bulk (Fig. 1A). The *xan-l.82* mutation has been identified previously and is located on chromosome 3H (Rzeznicka et al. 2005).

For whole genome sequencing, we aimed for 25 to 30× coverage using 150 bp paired-end sequencing. Sequencing yielded 444 and 633 million paired-end reads for the *xan-j.59* and *xan-l.82* bulks, respectively (Supplementary Table S1). To minimize noise due to incorrectly mapped reads, only reads with the maximum map quality were used for further analysis, which corresponded to approximately half of the raw data (Supplementary Table S1). This resulted in a sequencing depth of 17× and 24× for the *xan-j.59* and the *xan-l.82* bulks, respectively (Supplementary Table S2). After variant calling, SNPs within a depth of approximately 1 Sd from the average were retained for further analysis (Supplementary Fig. S1). This was done because regions of low coverage will result in noisy allele frequency calculations, and regions of high depth are likely to be artifacts due to mapping of similar repetitive sequences to the same position on the reference genome. To filter SNPs and map the location of the causal mutation, we used the allele depth difference calculated as the absolute value of the difference between read counts for 2 alleles divided by the sum of the read counts for the 2 alleles. This yields a value between 0 and 1. In each bulk, a value of 1 indicates that a SNP is completely linked to the phenotype in the analyzed data set. Since the 2 mutants, *xan-j.59* and *xan-l.82*, were both induced in the cultivar Bonus, most SNPs should be due to genetic differences between Bonus and Quench which is what allows the 2 bulks to serve as control samples for each other. To map one of the bulks, the SNPs were first filtered by removing loci with an allele depth difference greater than 0.3 in the control bulk as this was an indication that the SNP was not following Mendelian segregation and may thus be an artifact. Next, the allele depth differences were calculated for the bulk to be mapped. To denoise the data, a running median was calculated from the nearest 2,500 SNPs and plotted along each of the 7 chromosomes (Fig. 1B). This showed 2 small regions on chromosome 1H that were completely linked to the *xan-j.59* phenotype and 1 large region consisting of the recombination coldspot around the centromere on chromosome 3H that was completely linked to the *xan-l.82* phenotype.

The next step was to identify candidate genes in the linked genomic regions. BCFtools was used for variant calling to identify SNPs and small indels. If a causal mutation is not identified by traditional base callers or is expected to be a large genomic structural rearrangement, one could use specialized variant callers such as DELLY or BreakDancer to identify these even with short-read paired-end sequencing data (Chen et al. 2009; Rausch et al. 2012). Of the 1,426,075 alleles unique to the *xan-j.59* bulk, only 12,283 were homozygous (Table 1). The corresponding numbers for *xan-l.82* were 795,865 and 18,472. It was found that 4,202 and 10,940 of the homozygous alleles were in the mapped region of *xan-j.59* and *xan-l.82*, respectively (Table 1). Further analyses showed that only 2 and 8 of these alleles were located in high-confidence genes and caused changes of amino acid residues in the corresponding proteins (Table 1). The previously characterized magnesium protoporphyrin IX monomethyl ester cyclase gene was 1 of the 8 candidate genes for *xan-l.82*. Of the 2 candidates for *xan-j.59*, 1 was annotated as a SKI family transcriptional corepressor 1 and the other as chlorophyll synthase (HORVU.MOREX.r2.1HG0049060; Supplementary Table S3). As chlorophyll synthase is required for biosynthesis of chlorophyll and the identified mutation resulted in an early stop codon, this was a prime candidate.

**Table 1.** Filtration steps identifying candidate genes

Filtering level	*xan-j.59*	*xan-l.82*
Unique alleles on all chromosomes	1,426,075	795,865
Homozygous alleles on all chromosomes	12,283	18,472
Homozygous alleles on mapped chromosome	5,335	14,915
Homozygous alleles in mapped region	4,202	10,940
Homozygous alleles in mapped genes	33	36
Mapped genes with altered amino acid sequence	13	21
Mapped high-confidence genes with altered amino acid sequence	2	8

Unique alleles for each phenotypic bulk were generated by subtracting all alleles shared between the 2 samples. These were further filtered to narrow down the mutation causing the respective Xantha phenotype.

### Characterization of *xan-j.19*, *xan-j.59*, and *xan-j.64*

To validate that the correct gene had been identified, the chlorophyll synthase gene was PCR amplified and Sanger sequenced from *xan-j.59* as well as the other 2 allelic mutants *xan-j.19* and *xan-j.64*. The mutation identified from whole genome sequencing of the *xan-j.59* bulk was verified, and mutations were identified in *xan-j.19* and *xan-j.64*. Based on recommendations of the International Committee for Nomenclature and Symbolization of Barley Genes, the chlorophyll synthase gene is therefore named Xantha-j with the symbol *xan-j* (Franckowiak and Lundqvist 2019).

In *xan-j.19*, a 1 base pair deletion was identified in exon 13, and in *xan-j.64*, a single base pair substitution was found in exon 8 (Fig. 1C). The early stop codon in *xan-j.59* results in a peptide of 53 residues once the chloroplast transit peptide is removed while the mutation in *xan-j.64* results in an S212F substitution (Supplementary Fig. S2). The deletion in *xan-j.19* results in a frameshift, which causes the C-terminus of the protein to have 4 out of 5 incorrect residues after L340 followed by a premature stop codon (Supplementary Fig. S2).

**Figure 2.**
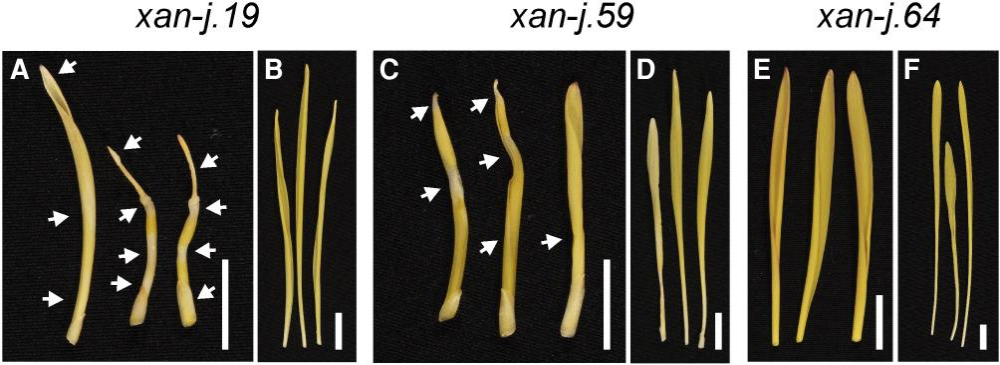
Seedling leaves of *xan-j* mutants grown in high light and low light. **A, C,** and **E)** Plants grown in greenhouse (approximately 10,000 lx). **B, D,** and **F**) Plants grown under a lab bench (approximately 100 lx). The seedling leaves of mutants *xan-j.19* and *xan-j.59* grown in high light show necrotic sections (indicated by arrows) corresponding to plant tissues developed during the night. No necrotic sections were observed if the plants were grown under low-light conditions. Mutant *xan-j.64* did not display any necrotic sections under high-light conditions. White bar, 1 cm.

**Figure 3.**
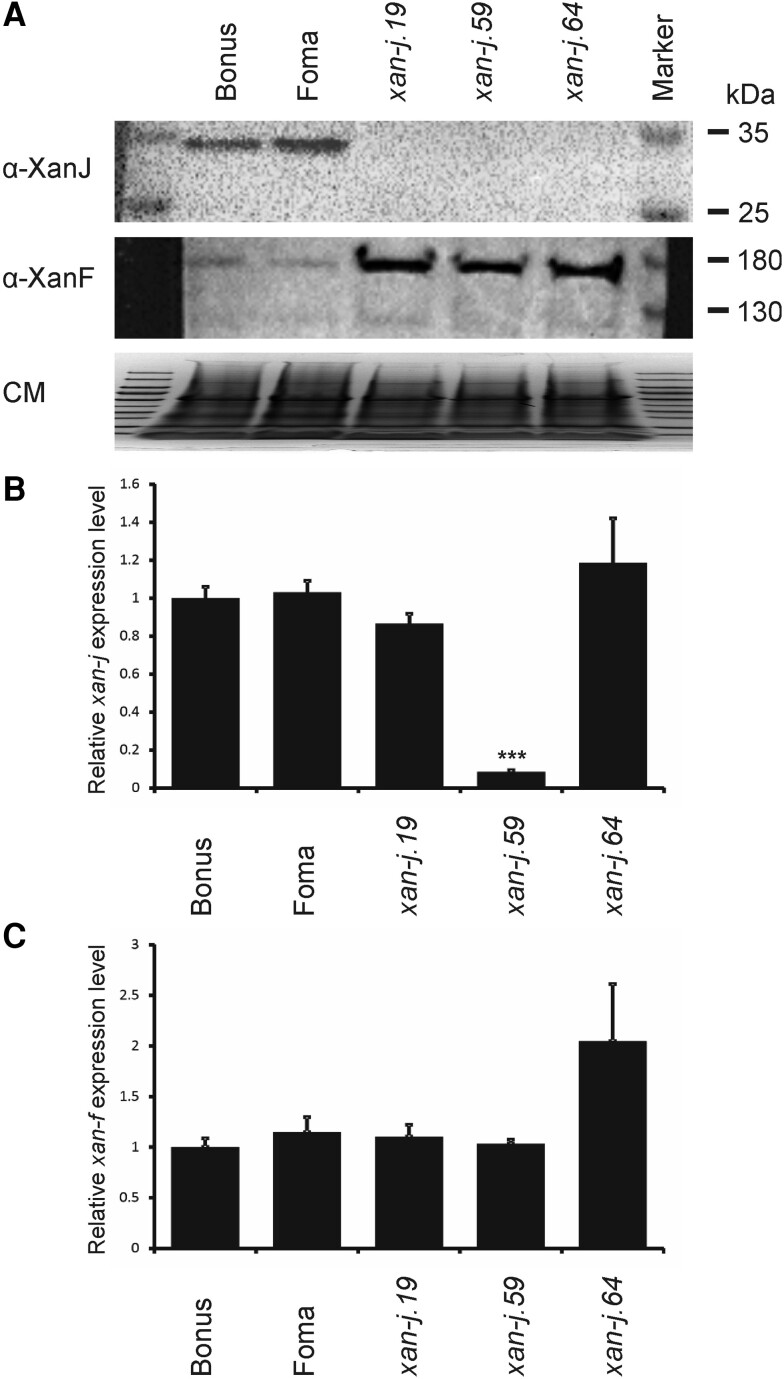
Presence of translation and transcription products in *xan-j* mutants. **A)** Immunoblot of *xan-j* mutants and the parental cultivars Foma and Bonus. All 3 mutants fail to accumulate XanJ. When probed with antibodies against the large subunit of the magnesium chelatase (XanF), the 3 *xan-j* mutants accumulate more XanF than the parental cultivars. As a loading control, a Coomassie-stained replica gel was run (CM). The Coomassie-stained image has been compressed. **B)** Relative expression of *xan-j*. **C)** Relative expression *of xan-f*. **B** and **C)** Expression levels are shown relative to Bonus and were determined by RT-qPCR from 4 biological replicates. Error bars show the standard error of the mean. A *t* test *P* < 0.001 is indicated by ***.

Although all 3 mutants have defects in chlorophyll synthase, the *xan-j.19* and *xan-j.59* mutants showed light sensitivity while *xan-j.64* did not. When grown under low light (approximately 100 lx), all 3 mutants were similar and produce yellow seedling leaves due to the lack of chlorophyll. However, when grown under high light (approximately 10,000 lx), *xan-j.19* and *xan-j.59* tend to form bleached sections similar to tigrina mutants, which show a striped phenotype due to accumulation of chlorophyll biosynthetic intermediates (Nielsen 1974; Hansson et al. 1997). The *xan-j.64* mutant showed no signs of damage when grown under high light (Fig. 2). The difference in light sensitivity among the *xan-j* mutants suggests that regulation of the chlorophyll biosynthesis pathway is affected differently depending on the nature of the mutation in *xan-j*. Since the terminal step of a biosynthetic pathway often produces feedback signals to key regulatory steps in the pathway, we analyzed protein and mRNA levels for chlorophyll synthase as well as the large subunit of the magnesium chelatase encoded by Xantha-f (*xan-f*) in barley (Olsson et al. 2004). The magnesium chelatase reaction is the first unique step of the chlorophyll biosynthetic pathway and has been identified as the main regulator for flux of protoporphyrin IX toward chlorophyll biosynthesis (Tanaka and Tanaka 2007). Interestingly, the amount of the large magnesium chelatase subunit was higher in all 3 *xan-j* mutants compared to the mother cultivars Bonus and Foma. The mRNA levels for *xan-f* were not significantly different in any of the mutants (Fig. 3). The XanJ protein was not detected in any of the 3 *xan-j* mutants while the mRNA levels were only significantly decreased in *xan-j.59* (Fig. 3), which has a nonsense mutation in the beginning of the gene. The mRNA level of *xan-j* had a slight but statistically nonsignificant decrease in *xan-j.19*, which has a nonsense mutation near the end of the gene due to the frameshift caused by the 1 base pair deletion.

### Natural variation in XanJ

Alleles affecting chlorophyll content may have an adaptive advantage in some natural or agricultural environments (Rotasperti et al. 2022). We therefore explored the natural variation in XanJ using an exome capture data set encompassing 815 barley cultivars, landraces, and wild barley lines (Chen et al. 2022b). Within this data set, we identified 241 variants within the chlorophyll synthase gene, of which 8 cause alterations of amino acid residues. All 8 changes were observed solely in wild barley lines, with half affecting residues within the chloroplast transit peptide (Supplementary Table S4). The most common of the variants, not found in the chloroplast transit peptide, was the substitution of Q343 with lysine. This allele was found in 19 out of 266 wild barley accessions. The remaining 3 alleles were much less common. I86T and V273I substitutions were found in 1 wild barley accession each, whereas an L290F substitution was found in 2 accessions. The 3 rare alleles were all found in accessions from the area around the Sea of Galilee (Supplementary Fig. S3). These alleles likely represent natural variation that was not retained during domestication since they are exclusively found in wild but not domesticated barley lines. They may have been lost due to selection if they underperformed or, more likely, they were simply not retained due to the bottleneck imposed by domestication. However, further analysis is required to determine whether these variations have a fine-tuning influence on chlorophyll content and photosynthetic efficiency.

### Modeling of an AlphaFold2-generated structure of chlorophyll synthase

No structure has been solved for chlorophyll synthase from any organism. Since artificial intelligence-based structural prediction is now able to generate high-quality models in silico, we constructed AlphaFold2 (Jumper et al. 2021a, 2021b) models for structural characterization of XanJ as well as variants affected by the mutations *xan-j.19* and *xan-j.64* with a truncated or modified (S212F) XanJ enzyme, respectively (Supplementary Fig. S4A). Most residues had a pLDDT (predicted local distance difference test) score above 90 (Supplementary Fig. S4B), which suggested that the AlphaFold2-generated models were of good quality. Residues with a pLDDT score above 90 generally have correct backbone placement and mostly correct side chain orientations (Tunyasuvunakool et al. 2021). Chlorophyll synthase belongs to the UbiA family of prenyltransferases (Cheng and Li 2014). Two structures have previously been solved for related prenyltransferase enzymes: AfUbiA from *Archaeoglobus fulgidus* (Huang et al. 2014) and ApUbiA from *Aeropyrum pernix* (Cheng and Li 2014). The structures were solved in their apo form as well as with bound geranyl pyrophosphate (GPP), dimethylallyl pyrophosphate (DMAPP; Huang et al. 2014), and geranyl thiolopyrophosphate (Cheng and Li 2014). Alignment of the XanJ model to the AfUbiA structure showed that the overall fold was conserved (Supplementary Fig. S5). The XanJ structure has 9 transmembrane helices connected by longer loops on the predicted stromal side and by comparatively short loops on the lumen side of the membrane (Supplementary Fig. S6).

**Figure 4.**
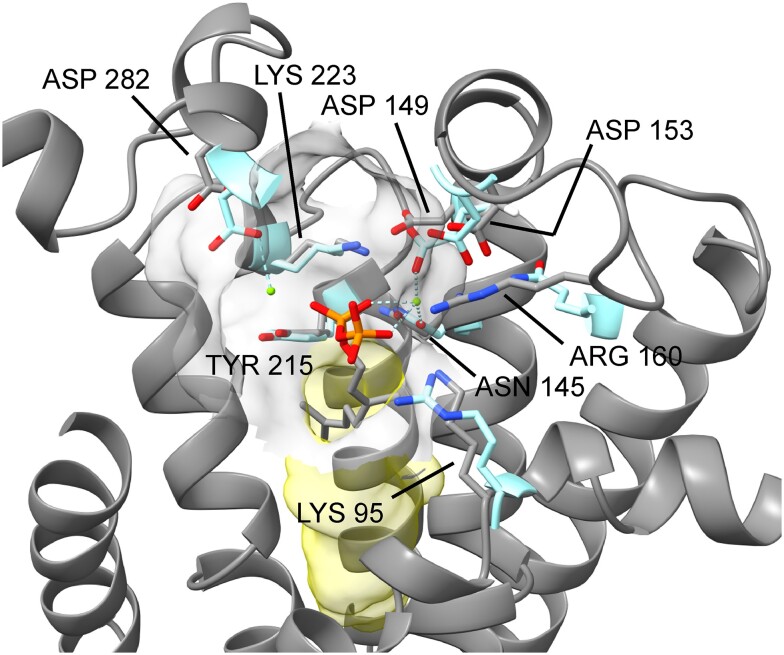
AlphaFold2 structural model of the globular cavity of XanJ. The pyrophosphate group of the prenyl substrate is located in the cavity (indicated in gray). The cavity contains several amino acid residues of importance for catalysis, substrate binding, or binding of a magnesium ion (green ball). The structural model has been aligned with the determined structure of AfUbiA containing a GPP. The corresponding amino acid residues of the AfUbiA structure are shown in turquoise. The PPP tunnel is shown in yellow. Red, oxygen; orange, phosphorus; blue, nitrogen.

The active site of XanJ must accommodate both substrates: chlorophyllide and GGPP/PhyPP. To identify the active site, we started by aligning XanJ to the GPP-bound AfUbiA structure. This placed GPP with the geranyl moiety in an elongated cavity with the pyrophosphate in a second more globular cavity. The globular cavity in turn connects to a distinctly flattened cavity near the pyrophosphate moiety (Supplementary Fig. S7). The elongated cavity of XanJ is likely to accommodate GGPP or PhyPP, which are twice as long as GPP, and will be referred to as the prenyl pyrophosphate (PPP) tunnel. The globular cavity housing the pyrophosphate contains 7 residues shown previously to be required for catalysis or binding of Mg^2+^ ions and prenyl substrates (Fig. 4; Cheng and Li 2014; Huang et al. 2014). Three residues are located in the end of 3 different transmembrane helixes or in short breaks of the helixes. The 4 other residues are found in loops on the stroma side of XanJ (Supplementary Table S5). The 7 residues have been studied by site-directed mutagenesis in various UbiA-related enzymes. In all cases, the modifications had severe effects on the enzymes (Supplementary Table S5). This also included N145, D149, D153, and R160 (XanJ numbering), which were studied in chlorophyll synthase from oat (*Avena sativa* L.; Schmid et al. 2002). Since the bonds between the pyrophosphate group and the prenyl moiety are broken followed by attachment of the prenyl group to chlorophyllide, the globular region around the phosphate should be closest to where catalysis takes place. We therefore refer to this region of the active site as the catalytic cavity.

**Figure 5.**
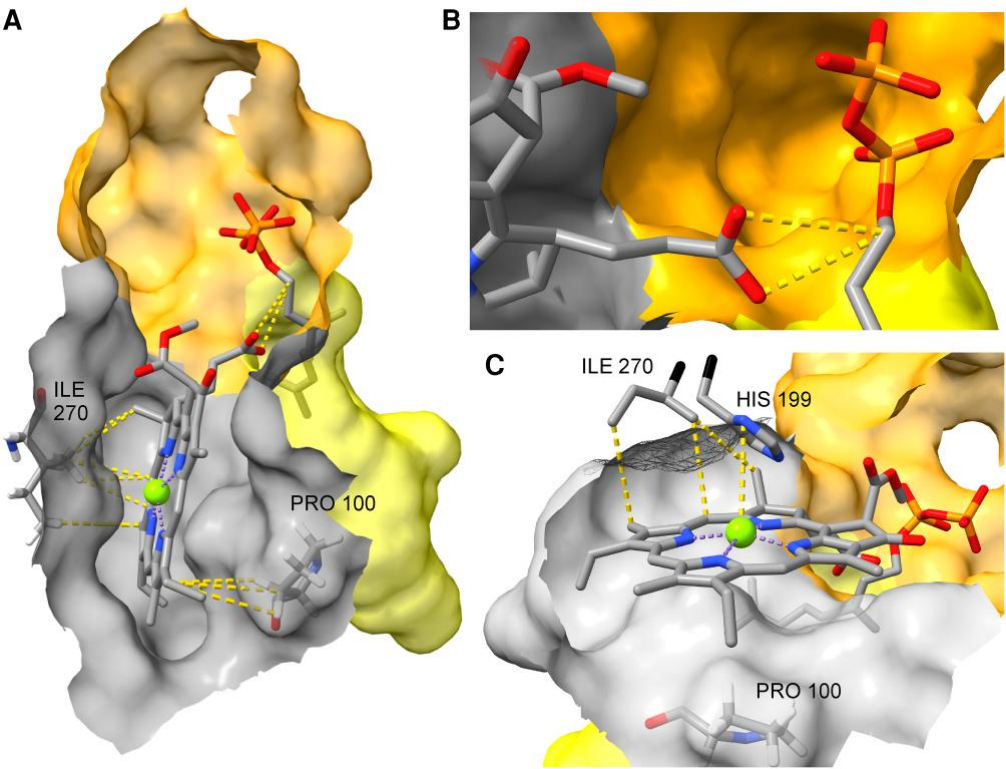
Chlorophyllide *a* modeled into the active site. **A)** Chlorophyllide *a* docked into the flattened pocket adjacent to the catalytic cavity (orange) and the PPP tunnel (yellow). **B)** The carbon-17 propionate group of chlorophyllide is close to the reactive GGPP carbon placed in the structure by alignment to AfUbiA. **C)** I270 is part of the tetrapyrrole-binding pocket. I270 is predicted to be in close proximity to the chlorophyllide based on studies of heme O synthase. The shown histidine residue (H199) is from structural alignment with *Bacillus subtilis* heme O synthase (AlphaFold Protein Structure Database AF-O31652-F1-model_v4). Red, oxygen; orange, phosphorus; blue, nitrogen; green, magnesium.

We next performed docking of chlorophyllide *a* to the XanJ structure using CB-Dock (Liu et al. 2020) to identify the tetrapyrrole-binding pocket. The CB-Dock engine first searches the enzyme surface for pockets and then uses AutoDock Vina to dock the substrate into the identified pockets (Trott and Olson 2010). This docked chlorophyllide into the flattened pocket, which is adjacent to the catalytic cavity. Furthermore, the docked orientation positioned the carbon-17 propionate group of chlorophyllide *a* with the carboxyl moiety in close proximity to the reactive GPP carbon placed in the structure by alignment to AfUbiA (Fig. 5).

Previous work has shown that switching between isoleucine and phenylalanine in *Rhodobacter sphaeroides* bacteriochlorophyll synthase (RsBchG) and *Synechocystis* chlorophyll synthase (SynChlG) at the position corresponding to P100 in XanJ alters the substrate specificity allowing RsBchG to utilize chlorophyllide *a* as a substrate and SynChlG to use bacteriochlorophyllide *a* (Kim et al. 2016). One would therefore expect P100 to be near a region of chlorophyllide *a* in which the structure differs from bacteriochlorophyllide *a*. Indeed, P100 is in close proximity to the carbon-8 ethyl group (Fig. 5). In chlorophyllide *a*, there is a double bond between carbon-7 and carbon-8, while in bacteriochlorophyllide *a*, this bond has been reduced to a single bond. Therefore, the methylene bridge of the ethyl group is in the same plane as the macrocycle of chlorophyllide *a* but not in bacteriochlorophyllide *a*.

**Figure 6.**
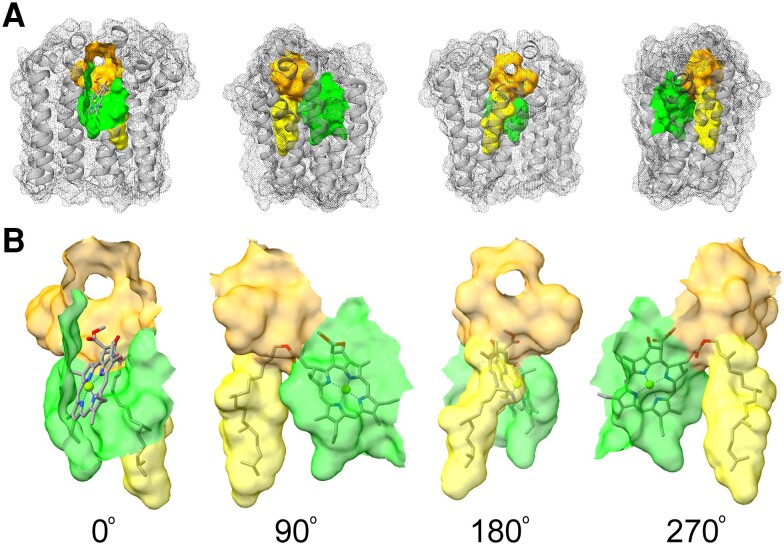
Suggested active site of barley chlorophyll synthase. The active site can be subdivided in the catalytic cavity (orange), the PPP tunnel (yellow), and the tetrapyrrole-binding pocket (green). The model is shown in 4 different views. Red, oxygen; blue, nitrogen; green spheres, magnesium. **A)** Overall view of the active site in the enzyme. **B)** Close-up of the active site with chlorophyll *a* docked to the model.

Support for correct substrate docking to XanJ can also be gained from heme O synthase, which is another prenyltransferase with a tetrapyrrole (heme) substrate. In heme O synthase, a histidine residue (H199) is proposed to be a ligand to Fe^2+^ of the heme substrate (Mogi 2009; Rivett et al. 2021). The corresponding residue is I270 in XanJ. In our XanJ structural model, I270 forms part of the surface of the flattened pocket where chlorophyllide *a* was predicted to bind (Fig. 5). We designate the flattened pocket as the tetrapyrrole-binding pocket. In summary, the active site can be subdivided into the PPP tunnel, the catalytic cavity, and the tetrapyrrole-binding pocket (Fig. 6).

### Evolutionary conserved amino acid residues

**Figure 7.**
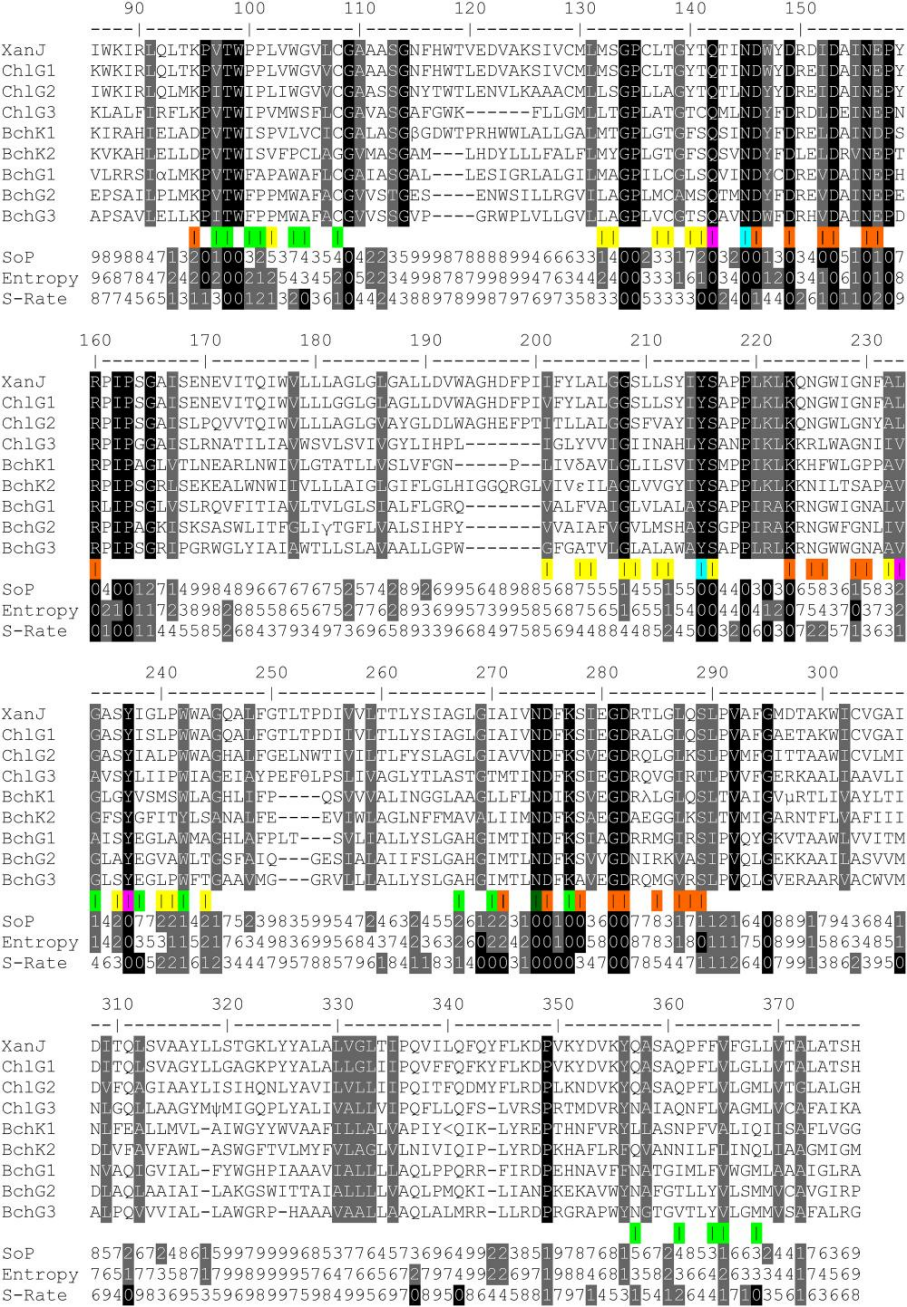
Protein sequence alignment of ChlG, BchG, and BchK. The alignment is based on 3,749 sequences that were clustered into 8 groups (Supplementary Fig. S8) to generate a consensus sequence representing each group. Positions with alternative amino acid residues have been indicated by Greek letters and symbols: α (A/T), β (R/Y), γ (I/L), δ (L/V), ε (G/S), θ (S/T), λ (K/Q), μ (K/R), ψ (F/L), and < (N/S). Amino acid residues forming the active site have been indicated by colors: orange (catalytic cavity), green (tetrapyrrole-binding pocket), yellow (GGPP tunnel), purple (all 3 subcompartments), turquoise (catalytic cavity and the GGPP tunnel), and dark green (catalytic cavity and the tetrapyrrole-binding pocket). Conserved and almost conserved residues are marked in black and gray, respectively. Three different conservation values were calculated for each position: SoP (sum of pairs according to the blosum62 scoring matrix); Entropy (based on amino acid frequencies), and S-Rate (site-specific substitution rate based on the phylogenetic tree). Zero represents the most conserved residues, and 9 represents the least conserved residues.

To further characterize functionally important residues, we retrieved and aligned 3,749 sequences of chlorophyll synthase (ChlG), bacteriochlorophyll synthase (BchG), and bacteriochlorophyll *c* synthase (BchK) from NCBI by blast. The alignment was trimmed to remove gaps relative to XanJ. We also trimmed residues before 41 since it has been shown that chlorophyll synthase from oat can be truncated in the N-terminus without loss of activity (Schmid et al. 2001). To cluster related sequences, a maximum likelihood tree was generated. From this, we designate 8 groups as ChlG1, ChlG2, ChlG3, BchK1, BchK2, BchG1, BchG2, and BchG3 (Supplementary Fig. S8). Consensus sequences were generated using the most frequent amino acid residue at each position for each of the 8 groups. These were aligned and shaded based on conservation in the consensus sequence alignment (Fig. 7). For each position in this alignment, we also calculated 3 different conservation measurements based on the full alignment. The first of these was site-specific substitution rates based on the phylogenetic tree since evolutionary rates will be slower for structurally or functionally critical residues. The other 2 measures were sum of pairs using the blosum62 scoring matrix and entropy (Pei and Grishin 2001; Pettersen et al. 2021). The conservation values were grouped into 10 quantiles where 0 represents the most conserved and 9 represents the least conserved residues. The conserved amino acid residues were viewed in the light of the Alphafold2-generated structural model of XanJ. As might be expected, conservation was highest at and around residues forming the active site of the enzyme with clearly higher conservation of residues of the catalytic cavity compared to the tetrapyrrole-binding pocket and the PPP tunnel (indicated by orange, green, and yellow, respectively, in Fig. 7).

Two completely conserved residues, Q142 and Y237, and 1 semiconserved residue, L233, are part of all 3 subcompartments (purple in Fig. 7). Inspection of the chlorophyllide *a* docked XanJ model suggests that Q142 and Y237 are hydrogen bond donors to the carbon-17 propionate oxygen which is the site of bond formation during catalysis (Supplementary Fig. S9). Of note, Y237 is located in a break of transmembrane helix 5 (TMH5) and the aromatic ring is placed in-between the tetrapyrrole-binding pocket and the PPP tunnel such that hydrophobic interactions between both substrates and Y237 are possible. The position of L233 is generally occupied by a nonpolar residue and is likely to create a hydrophobic patch for interaction with ring D of chlorophyllide (Supplementary Fig. S9). The methyl group from a completely conserved residue, T98, forms part of the hydrophobic surface near the carbon-17 propionate carbon and is within contact distance of the side chains of Q142 and Y237 (Supplementary Fig. S9). Additionally, the backbone and the side chain hydroxyl group of T98 are H bond donors to backbone carbonyl oxygen of K95 present in the catalytic cavity, which has been shown to be essential for PPP binding in other UbiA family enzymes (Cheng and Li 2014; Huang et al. 2014).

The residues that form the tetrapyrrole-binding pocket have predominantly hydrophobic side chains (green in Fig. 7). However, some of the enzyme clusters have positions with conservation of charged or polar residues, which is likely because some of the differences between the different tetrapyrrole substrates is the presence or absence of oxygen on side groups. For example, position 238 has a completely conserved glutamate residue in BchG, which might bind the carbonyl oxygen present on carbon-2 of bacteriochlorophyllide *a* via a bridging water molecule. The residues N274 and K277 are completely conserved. K277 is located in loop 6-7 and is likely able to undergo relatively large positional changes during the catalytic cycle. Although speculative, 1 suggestion is that it may move far enough to act as an H bond donor to the ring E carbonyl oxygen of the chlorophyllide, which would likely also seal off the active site during catalysis. N274 is located just at the junction of transmembrane helix 6 and loop 6-7. Slight motion could place the side chain amide group in the catalytic cavity. The corresponding position in ApUbiA, Y178, has been implicated in decreased PPP binding when substituted for alanine (Cheng and Li 2014).

In general, the catalytic cavity has the most highly conserved residues. In addition to the residues shown in Fig. 5, positions corresponding to D146, N156, E157, D275, and S289 are highly conserved as charged or polar residues capable of hydrogen bonding. These residues are likely also essential for interactions with Mg^2+^ ions or the pyrophosphate group during the catalytic cycle.

**Figure 8.**
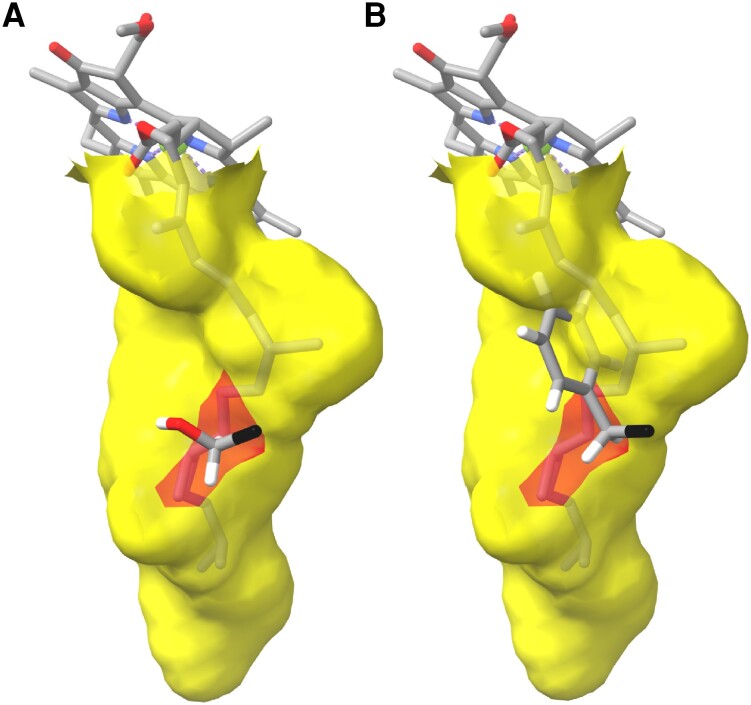
The surface of the PPP tunnel (yellow) with a chlorophyll *a* molecule docked in the active site. Red, oxygen; blue, nitrogen; white, hydrogen; black, the carboxyl carbon of the amino acid residue. **A)** S212 contributes to the surface of the PPP tunnel. **B)** In mutant *xan-j.64*, S212 has been changed to a phenylalanine. A steric clash occurs between the bulky side chain of phenylalanine and the phytol moiety.

Residues of the PPP tunnel tend to have hydrophobic side chains but otherwise most positions are fairly variable (yellow in Fig. 7). One position, S216, is completely conserved. In XanJ, the hydroxyl group is likely able to act as an H bond donor to the backbone oxygen of S212 and an H bond acceptor from side chain of N145 (turquoise in Fig. 7). S212 is exchanged to phenylalanine by the mutation in *xan-j.64*, and N145 is a conserved residue that is part of the catalytic cavity as well as the PPP tunnel. S212 contributes to the surface of the PPP tunnel and is conserved as small amino acid residues. The AlphaFold2-generated model of XanJ.64 clearly shows a steric clash between the bulky side chain of phenylalanine at position 212 and the isoprenoid substrate (Fig. 8).

### Prenyl substrate is required for in vivo chlorophyll synthase accumulation

**Figure 9.**
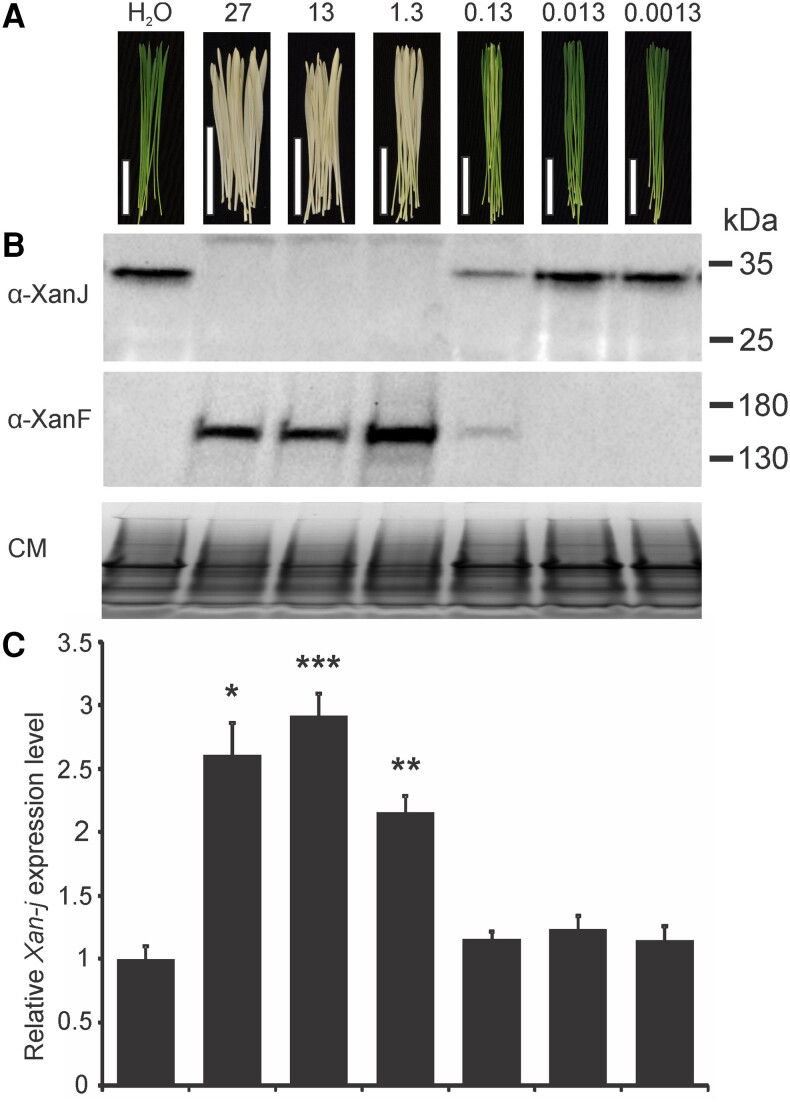
Effect of clomazone treatment on growing barley seedlings. **A)** Visual effect of clomazone treatment on barley seedlings. The concentrations are in milligrams per liter. White bar, 5 cm. **B)** Immunoblot analyses showed that XanJ was absent in seedlings where clomazone treatment had generated a white phenotype. In contrast, the large subunit of the magnesium chelatase (XanF) was more abundant in the same plants. As a loading control, a Coomassie-stained replica gel was run (CM). The Coomassie-stained image has been compressed. **C)** RT-qPCR revealed an increased expression of the *xan-j* gene in clomazone-affected seedlings. Expression levels are shown relative to water-treated plants from 4 biological replicates. Error bars show the standard error of the mean. A *t* test *P* < 0.001 is indicated by ***, *P* < 0.01 by **, and *P* < 0.1 by *.

The effect of the *xan-j.64* mutation on the enzyme structure suggests that it may prevent GGPP/PhyPP binding to chlorophyll synthase, and we could not detect the enzyme in the mutant (Fig. 3). To test if binding of the isoprenoid substrate may be required for stable maintenance of chlorophyll synthase in vivo, we induced GGPP/PhyPP deficiency by treating wild-type plants with clomazone (2-[(2-chlorophenyl)methyl]-4,4-dimethyl-3-isoxazolidinone). Clomazone is an herbicide commonly used in agriculture. Clomazone has a dramatic effect on chloroplast pigmentation and results in white or pale green plants, depending on the amount applied (Duke and Kenyon 1986; Duke and Paul 1986). In vivo, clomazone is oxidized to 5-ketoclomazone (Ferhatoglu et al. 2005). 5-Ketoclomazone inhibits deoxyxylulose-5-phosphate synthase, which is the first enzyme of the nonmevalonate isoprenoid synthesis pathway (Ferhatoglu and Barrett 2006). This prevents the biosynthesis GGPP/PhyPP, which in turn prevents synthesis of carotenoids and chlorophylls. A clear doze response was seen with treated seedlings being white, pale green, or green depending on clomazone concentration (Fig. 9). We noted that there were no obvious visual signs of light sensitivity in clomazone-treated plants. We analyzed the treated plants for the presence of *xan-j* and *xan-f* gene products. In the white plants with the highest clomazone treatments, no chlorophyll synthase could be detected and there was an increased amount of the large magnesium chelatase subunit (Fig. 9). This is similar to the situation observed in the *xan-j* mutants. RT-qPCR analyses showed a statistically significant increase in the levels of *xan-j* mRNA in the white plants (Fig. 9).

## Discussion

The availability of barley genome sequence resources (Mascher et al. 2017; Monat et al. 2019) has provided a renaissance for the thousands of barley mutants available at various gene banks and seed stores around the world, since they provide excellent opportunities for functional studies of genes. By connecting a gene to a mutant phenotype, the function of the gene is revealed. In the present study, we used 2 chlorophyll-deficient barley mutants and developed a pipeline for identifying causal mutations. We utilized only standard bioinformatic tools, which should be available on most computing clusters, and relatively simple calculations for the allele depth difference. The methodology should be easy for others to adopt and adapt.

After creating a mutant population, researchers or plant breeders are likely to have many mutants generated in the same genetic background that are of scientific or applied interest, which they would like to identify. The strategy of using 2 different populations of interest with the same genetic background as controls for each other effectively reduces costs by 50%, regardless of species or sequencing technology used. Further, our results can be used as a guideline for experiment design and as control data by other researchers for identification of causal mutations in future studies where the genetic background of the mutant is the cultivar Bonus. This includes the approximately 3,000 classical mutants induced in Bonus that are curated at the Nordic Genetic Resource Center (www.nordgen.org) in Alnarp, Sweden.

Mutations located in recombination coldspot regions have traditionally been difficult to identify by map-based cloning, which relies on genetic recombination to narrow down the genomic interval until it is small enough to contain a low number of candidate genes. As a proof of principle to show that our methodology could efficiently be used for identifying mutations in recombination-poor regions, the previously characterized *xan-l.82* mutant was included in this study, which is known to be located in the centromeric region of chromosome 3H (Rzeznicka et al. 2005). The final list of candidates contained only 8 genes, one of which was correct, i.e. the gene encoding magnesium protoporphyrin IX monomethyl ester cyclase with a TGG to TGA nonsense mutation (W291 to premature stop; Rzeznicka et al. 2005). The chromosomal location of *xan-j* was previously unknown but found to be in the telomeric region of chromosome 1H. From analyses of the *xan-j.59* bulk, the candidate list was only 2 genes including the chlorophyll synthase gene. Thus, from 795,865 and 1,426,075 identified unique alleles in the *xan-l.82* and *xan-j.59* pools, respectively, it was possible to generate a very short list of candidate genes.

Chlorophyll synthase has been studied in multiple organisms such as *Synechocystis* (Oster et al. 1997), oat (Schmid et al. 2001, 2002), rice (*Oryza sativa* L.; Wu et al. 2007), and tobacco (*Nicotiana tabacum* L.; Shalygo et al. 2009; Kim et al. 2023). Two of the studies also contained comparisons to the closely related BchG of *Rhodobacter capsulatus* and *R. sphaeroides* (Oster et al. 1997; Kim et al. 2023). To further advance the knowledge about the enzymatic mechanism, structural information about the proteins would be most helpful. However, chlorophyll synthase is an intrinsic membrane protein, which has hampered such efforts. Recently, AlphaFold2 was published as a machine learning approach for structure prediction with unprecedented accuracy (Jumper et al. 2021a). We used AlphaFold2 to develop a 3D structure of the barley chlorophyll synthase. Like 2 related prenyltransferases, AfUbiA and ApUbiA, derived from 2 archaea, the chlorophyll synthase has 9 transmembrane-spanning helixes. The UbiA structures with bound PPP molecules helped us to identify the active site of the chlorophyll synthase, which consists of a tetrapyrrole-binding site and a PPP tunnel, which are connected to a catalytic cavity where the propionate group of carbon-17 of chlorophyllide is in close proximity to the reactive carbon of GGPP/PhyPP. A multisequence alignment involving 3,749 sequences of chlorophyll and BchGs highlighted many conserved amino acid residues. The function of most of the conserved residues could be understood from their location in the 3D structure.

The *xan-j.59* mutant has an early nonsense mutation that results in no detectable XanJ protein on western blots as well as decreased mRNA levels, likely due to nonsense-mediated decay (Raxwal and Riha 2023). Similarly, the *xan-j.19* mutant also has a premature stop codon, but the stop is close to the end of the gene and the *xan-j* mRNA level was not significantly decreased. Probably the effect of nonsense-mediated decay is smaller with the mutation near the end since more of the mRNA would be protected by translating ribosomes. Since the mRNA level in *xan-j.19* is clearly close to that of wild type, it is likely that a truncated protein is produced and then degraded since the enzyme was not detected on western blots. Although the mutation is near the end of the gene, it alters or removes 36 of the 37 terminal amino acid residues. These changes affect 2 transmembrane helices, one of which is completely missing. It is likely that such a structural change prevents proper folding. Cellular machinery would likely detect this as a damaged protein and degrade it accordingly. The *xan-j.64* mutant also did not accumulate the XanJ protein while mRNA levels were unchanged. Thus, the amino acid substitution is also likely to cause the enzyme to be degraded in planta.

The absence of XanJ protein according to western blot analyses in all 3 mutants makes it hard to understand why the *xan-j.19* and *xan-j.59* mutants are sensitive to high-light intensity while *xan-j.64* is not. Possibly, a small amount of XanJ protein is still present in *xan-j.64*, although not detectable by western blot, which affects the regulation of the chlorophyll biosynthetic pathway. However, all 3 *xan-j* mutants showed an equally increased level of the large magnesium chelatase subunit XanF, which is at the main regulatory point for directing tetrapyrrole precursors toward chlorophyll biosynthesis. Thus, the magnesium chelatase levels alone are not likely to be the main regulatory variable explaining the difference in light sensitivity. However, the tetrapyrrole biosynthesis pathway is also regulated posttranslationally (Wang et al. 2022) so the in vivo magnesium chelatase activity may be different between the light sensitive and insensitive *xan-j* mutants even if enzyme levels are similar. The other main regulatory point affecting chlorophyll precursor levels is the formation of aminolevulinic acid (ALA), which is the first committed step for tetrapyrrole biosynthesis (Tanaka and Tanaka 2007). A previous study on a rice chlorophyll synthase mutant with decreased enzymatic activity showed increased levels of ALA, protoporphyrin IX, Mg-protoporphyrin IX, and protochlorophyllide, as well as chlorophyllide, suggesting that regulation may be affecting the ALA synthesis step, although they noted decreased mRNA levels for glutamyl-tRNA reductase and the increased precursor levels might simply be due to the decreased conversion of chlorophyllide *a* to chlorophyll *a* (Wu et al. 2007). However, another study on tobacco chlorophyll synthase antisense lines revealed a tendency for decreased levels of intermediates as well as enzymatic activity of ALA formation and the early steps of the chlorophyll branch, suggesting that the pathway was downregulated by decreased chlorophyll synthase levels (Shalygo et al. 2009). The same study observed the opposite tendency in tobacco chlorophyll synthase overexpression lines. The inconsistencies between these 2 studies may be due to species differences or have to do with the mechanism behind how chlorophyll synthase was affected. In the rice study, the enzymatic activity of chlorophyll synthase was reduced in vitro due to the identified mutation while in vivo enzyme levels were not determined. In the tobacco study, chlorophyll synthase levels were reduced by RNAi but the function of the enzyme itself was not altered due to mutation. Neither of the 2 studies looked at a situation where there was essentially a complete block of chlorophyll synthase activity. The 3 barley *xan-j* mutants all result in a more severe block of the chlorophyll synthase step and do not accumulate chlorophyll synthase in vivo, yet they have differing light sensitivity phenotypes. We therefore looked at AlphaFold2 models of the *xan-j.64* mutant protein for clues as to how the S212F modification may affect chlorophyll synthase function. Although S212 is not strictly conserved, it is conserved as a small residue (serine, alanine, or glycine) and is located in the PPP tunnel. We postulate that a bulky phenylalanine instead of S212 abolishes binding of GGPP/PhyPP to the active site of chlorophyll synthase. In order to analyze if the absence of XanJ in the *xan-j.64* mutant is connected to a requirement of GGPP/PhyPP binding, we exposed growing barley plants to clomazone, which is an inhibitor of chloroplastic isoprenoid biosynthesis (Ferhatoglu and Barrett 2006). This prevents synthesis of GGPP/PhyPP. We found that chlorophyll synthase was absent in clomazone-treated plants. Thus, absence of GGPP/PhyPP binding in both the mutant *xan-j.64* and clomazone treatment of barley seedlings suggested that there might be a requirement of GGPP/PhyPP binding to the active site of chlorophyll synthase to stabilize the enzyme. Furthermore, *xan-j* mRNA levels were increased by clomazone treatment even though the protein was not detected. This suggests that either translation of the mRNA is repressed or more likely that the chlorophyll synthase protein is rapidly degraded following translation. The levels of XanF were increased by clomazone treatment, which is similar to the increase in the *xan-j* mutants. Since chlorophyll synthase needs to coordinate chlorophyll synthesis with isoprenoid synthesis, the enzyme may act as a GGPP/PhyPP sensor by requiring the bound isoprenoid substrate for stability. In the AfUbiA structure, it was noted that substrate binding resulted in an unstructured loop region becoming ordered (Huang et al. 2014). A similar observation was made with the ApUbiA structure, which resulted in a loop region becoming ordered and thereby protected from protease degradation upon binding the prenyl substrate (Cheng and Li 2014). These loop regions correspond to regions of chlorophyll synthase that are likely to be exposed to the stroma, and conformational changes could expose a degron to stromal proteases when chlorophyll synthase is not bound to GGPP/PhyPP. The lack of a light-sensitive phenotype of *xan-j.64* further suggests that the mechanism of chlorophyll synthase degradation due to lack of the isoprenoid substrate might result in a signal to downregulate flux through the chlorophyll synthesis pathway. This signal does not seem to be generated if chlorophyll synthase is degraded due to damage as in *xan-j.19* or completely missing as in *xan-j.59*. Thus, it is unlikely that this signal is simply the lack of chlorophyll synthase. The mammalian prenyltransferase UBIAD1, which utilizes GGPP for vitamin K synthesis, has been shown to act as a GGPP sensor to modulate flux through the cytosolic mevalonate pathway for isoprenoid biosynthesis (Nakagawa et al. 2010; Schumacher et al. 2015). When sterols bind to 3-hydroxy-3-methylglutaryl coenzyme A reductase (HMGCR), which catalyzes the committed step of the mevalonate isoprenoid biosynthetic pathway, HMGCR is targeted for degradation by ubiquitination and binds UBIAD1. HMGCR is protected from degradation by binding UBIAD1. When UBIAD1 binds GGPP, it releases HMGCR that can then be degraded. UBIAD1 thus acts as a GGPP sensor to decrease flux through the mevalonate pathway when 2 different end products, sterols and GGPP, are present. The interaction between HMGCR and UBIAD1 is mediated by interactions between transmembrane helices on both proteins, and it seems that this interaction is only disrupted by supplying geranylgeraniol and not farnesol (Schumacher et al. 2015; Chen et al. 2022a). Induced regulatory conformational changes in membrane regions or surface-exposed regions by isoprenoid substrate binding to UbiA family proteins thus seem to be a common feature (Nakagawa et al. 2010; Cheng and Li 2014; Huang et al. 2014; Schumacher et al. 2015; Chen et al. 2022a). A GGPP/PhyPP sensing role for chlorophyll synthase is also consistent with the enzymatic mechanism of the chlorophyll synthase. The enzyme has previously been shown to follow a ping-pong mechanism with the isoprenoid substrate binding first and catalysis occurring upon chlorophyllide binding (Schmid et al. 2002). Since chlorophyll precursors are phototoxic, accumulation must be avoided. A mechanism where the chlorophyll biosynthetic pathway is downregulated when isoprenoid synthesis is lacking is thus necessary to avoid phototoxic effects. In the clomazone-treated plants, chlorophyll synthase did not accumulate. However, there was an increased expression of *xan-j*. This suggests that the plants are poised to resume chlorophyll synthesis when GGPP/PhyPP becomes available. It is tempting to speculate that in this state, there is a high turnover of chlorophyll synthase in a GGPP/PhyPP unbound confirmation with steady-state levels of chlorophyll synthase under the detection limit of our immunoblots. This population of chlorophyll synthase molecules may be the origin of the regulatory signal. Future studies will need to elucidate the molecular mechanism by which chlorophyll synthase acts as a GGPP/PhyPP sensor.

## Materials and methods

### Plant material

The barley (*H. vulgare* L.) mutants *xan-j.19*, *xan-j.59*, *xan-j.64*, and *xan-l.82* belong to the chlorophyll mutant collection previously kept at the Carlsberg Research Laboratory (Hansson 2007). Mutants *xan-j.19*, *xan-j.59*, and *xan-l.82* were derived from cultivar Bonus mutant populations in 1954, 1956, and 1975, respectively, whereas *xan-j.64* was induced in Foma in 1958. Mutant *xan-j.19* was induced by X-rays, *xan-j.59* by p-*N*-di(β-chloroethyl)phenylalanine, *xan-j.64* by diethyl sulfate, and *xan-l.82* by sodium azide (Henningsen et al. 1993). The mutants are kept as heterozygous stocks due to the lethal phenotype of the mutations. Mapping populations were constructed by crossing the *xan-j.59* and *xan-l.82* mutants to the cultivar Quench as the male parent.

### Genotyping of F_2_ mapping populations

Genomic DNA was isolated from leaves of pooled F_2_ plants by a modified CTAB protocol as previously described (Stuart et al. 2021). Genomic DNA sequencing was performed by SciLifeLab (scilifelab.se) using an Illumina NovaSeq 6000 with 150 paired-end cycles. The library preparation was also performed by SciLifeLab using the TruSeq PCR-free DNA library preparation kit (Illumina Inc.). Sequencing reads were aligned to the barley MorexV2 reference genome using BWA mem (version 0.7.17; Li and Durbin 2010; Monat et al. 2019). Samtools fixmate (samtools version 1.10) was used to fill in mate coordinates. BCFtools (BCFtools version 1.17) was used for variant calling with flags -q 60 -Q 30 -D (Li 2011). For mapping, only biallelic SNPs present in both bulks were used. Allele depth differences were calculated in UNIX using awk. The zoo package was used for calculating running ADD medians and plotted with ggplot in R version 4.3.1. To identify alleles observed in a mutant sample and not found in the control, the BCFtools contrast plugin was used. BCFtools was used to filter unique variants. Functional annotation of variants was performed with snpEff version 4.3t.

50k SNP genotyping was conducted at the James Hutton Institute, Dundee, United Kingdom, using the barley 50k iSelect SNP chip (Bayer et al. 2017). Flapjack (Milne et al. 2010) was used for genotypic visualization of the 50k data.

### General DNA techniques

Genomic DNA for PCR reactions was extracted from fresh leaves using the REDExtract-N-Amp Plant PCR Kit (Sigma-Aldrich, St. Louis, MO, United States). PCRs were performed by initial denaturation at 94 °C/3 min, followed by 35 cycles of 94 °C/30 s, 60 °C/30 s, and 72 °C/60 s, with a final extension step of 72 °C/5 min. Purification of PCR products was done by using Illustra ExoProStar 1-Step (Cytiva, Marlborough, MA, United States). Sanger sequencing was performed by Eurofins Genomics, Germany. RT-qPCR was performed from isolated total RNA as previously described (Stuart et al. 2021). Primers used in this study are listed in Supplementary Table S6. Expression levels were determined by RT-qPCR from 4 biological replicates as previously described (Stuart et al. 2021). Significant differences were calculated using a 2-sided Student's *t* test.

### Extraction of natural variation in XanJ

SNPs in the XanJ region (XanJ: HORVU1Hr1G059890 chr1H:435325093-435331638) were extracted from 815 exome sequenced cultivars, landraces, and wild barleys (Chen et al. 2022b) using BCFtools (Li 2011). Google My Maps was used to visualize the origin of barley accession.

### Modeling of a XanJ 3D structure

AlphaFold version 2.1.1 (Jumper et al. 2021a, 2021b) was used to construct a 3D structural model of the *xan-j* gene product without the chloroplast transit peptide. The required databases (BFD, mgnify, pdb70, pdb_mmcif, small_bfd, uniclust30, and uniref90) for structural prediction were downloaded on September 22, 2021. Structures were visualized with UCSF ChimeraX (Pettersen et al. 2021).

### Chlorophyll synthase residue conservation

Sequences were aligned with Clustal Omega version 1.2.4 (Sievers and Higgins 2018). IQ-Tree version 2.2.2.2 was used to determine the best fit model as LG + F + I + R10, which was used to infer the phylogenetic tree and site-specific substitution rates (Minh et al. 2020). Consensus sequences of phylogenetic clusters were defined as the most frequent amino acid at each position and calculated with BioEdit version 7.2.5 and Excel (Hall 1999). Conservation measures sum of pairs and entropy were calculated with AL2CO as implemented in ChimeraX (Pei and Grishin 2001; Pettersen et al. 2021).

### Western blot

Extraction of total proteins from barley leaves, separation of protein by SDS–PAGE, and immunoblots were performed as described previously (Stuart et al. 2020, 2021). Polyclonal rabbit (*Oryctolagus cuniculus* L.) antibodies against barley XanJ were generated by Agrisera (Vännäs, Sweden) using synthetic peptides as antigen. The following synthetic peptides, derived from the XanJ amino acid sequence, were used: CAKQEDNIWKIRLQL and YDRDIDAINEPYRPIPC.

### Accession numbers

Sequence data from this article can be found in the NCBI Sequence Read Archive (https://www.ncbi.nlm.nih.gov/sra) under accession number PRJNA1095161.

## Supplementary Material

kiae218_Supplementary_Data

## Data Availability

The data underlying this article are available in the article, in its online supplementary material, and in the NCBI Sequence Read Archive (https://www.ncbi.nlm.nih.gov/sra) under BioProject accession number PRJNA1095161.

## Supplementary data

The following materials are available in the online version of this article.

**Supplementary Figure S1**. The frequency distribution of sequencing depth for called SNPs.

**Supplementary Figure S2**. Alignment of barley XanJ and the polypeptides resulting from the mutations in the 3 mutants *xan-j.19*, *xan-j.59*, and *xan-j.64.*

**Supplementary Figure S3**. Geographical distribution of barley lines with variation in the chlorophyll synthase gene *xan-j.*

**Supplementary Figure S4**. Structural prediction of barley XanJ.

**Supplementary Figure S5**. Alignment of the XanJ model to the UbiA structure of *A. fulgidus* determined by X-ray crystallography.

**Supplementary Figure S6**. AlphaFold-generated structural model of barley XanJ.

**Supplementary Figure S7**. The GPP in the active site of XanJ after alignment of XanJ to the GPP-bound AfUbiA structure.

**Supplementary Figure S8**. Maximum likelihood phylogenetic tree.

**Supplementary Figure S9**. Position of 5 key amino acid residues in the active site of barley XanJ.

**Supplementary Table S1**. Number of sequence reads.

**Supplementary Table S2**. Sequence coverage and depth.

**Supplementary Table S3**. Candidate genes containing homozygous mutations in the mapped chromosomal regions.

**Supplementary Table S4**. Natural allelic variation in *xan-j* according to a panel of 815 barley cultivars, landraces, and wild barley lines.

**Supplementary Table S5**. Summary of amino acid residues located in the active site of XanJ and/or previously studied in related enzymes.

**Supplementary Table S6**. Primers used for PCR and RT-qPCR amplifications of *xan-j.*

## Dive Curated Terms

The following phenotypic, genotypic, and functional terms are of significance to the work described in this paper:

Chlorophyll CHEBI: CHEBI:28966

GPP CHEBI: CHEBI:17211

ALA CHEBI: CHEBI:17549
