# Supplementary material for: A pipeline for identification of causal mutations in barley identifies Xantha-j as the chlorophyll synthase gene
Source: Plant Physiol. 2024 Apr 17;195(4):2877–90. doi: 10.1093/plphys/kiae218 (PMC11288739; doi:10.1093/plphys/kiae218)
Supplement: kiae218_Supplementary_Data [file kiae218_supplementary_data.zip › Supplementary Data 1.pdf]

SUPPLEMENTARY FIGURES

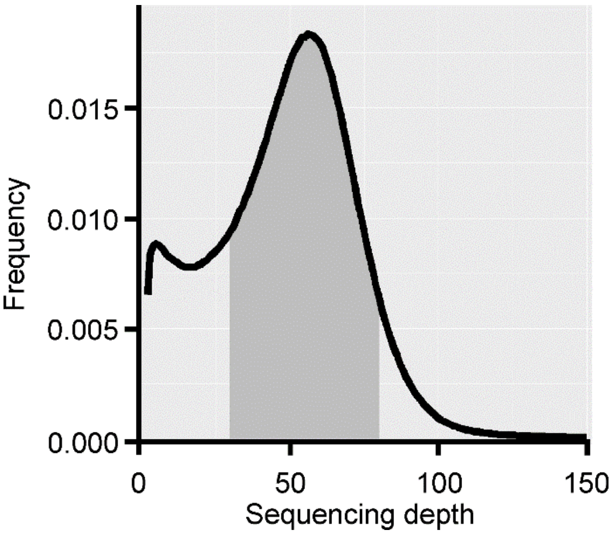

Supplementary Figure S1. The frequency distribution of sequencing depth (number of reads covering each SNP) for called SNPs. The shaded area corresponds to SNPs with a sequencing depth of 31x-80x for the two phenotypic bulks combined and represents 69% of the data or approximately one standard deviation from a normal distribution. These numbers divided by 2 were used for filtering the minimum and maximum sequencing depth SNPs for each phenotypic bulk.

```
xan-j    MATSHFLAAAAATSSSSTAFRPPLRFLSLPPPSLTLSRRRPFPVVCAADADAKETTKPKV 60
xan-j.19 MATSHFLAAAAATSSSSTAFRPPLRFLSLPPPSLTLSRRRPFPVVCAADADAKETTKPKV 60
xan-j.59 MATSHFLAAAAATSSSSTAFRPPLRFLSLPPPSLTLSRRRPFPVVCAADADAKETTKPKV 60
xan-j.64 MATSHFLAAAAATSSSSTAFRPPLRFLSLPPPSLTLSRRRPFPVVCAADADAKETTKPKV 60

xan-j    PEKAPAAGSSFNQLLGIKGAKQEDNIWKIRLQLTKPVTWPPLVWGVLCGAAASGNFHWTV 120
xan-j.19 PEKAPAAGSSFNQLLGIKGAKQEDNIWKIRLQLTKPVTWPPLVWGVLCGAAASGNFHWTV 120
xan-j.59 PEKAPAAGSSFNQLLGIKGAKQEDNIWKIRLQLTKPVT---------------------- 98
xan-j.64 PEKAPAAGSSFNQLLGIKGAKQEDNIWKIRLQLTKPVTWPPLVWGVLCGAAASGNFHWTV 120

xan-j    EDVAKSIVCMLMSGPCLTGYTQTINDWYDRDIDAINEPYRPIPSGAISENEVITQIWVLL 180
xan-j.19 EDVAKSIVCMLMSGPCLTGYTQTINDWYDRDIDAINEPYRPIPSGAISENEVITQIWVLL 180
xan-j.59 ------------------------------------------------------------ 98
xan-j.64 EDVAKSIVCMLMSGPCLTGYTQTINDWYDRDIDAINEPYRPIPSGAISENEVITQIWVLL 180

xan-j    LAGLGLGALLDVWAGHDFPIIFYLALGGSLLSYIYSAPPLKLKQNGWIGNFALGASYIGL 240
xan-j.19 LAGLGLGALLDVWAGHDFPIIFYLALGGSLLSYIYSAPPLKLKQNGWIGNFALGASYIGL 240
xan-j.59 ------------------------------------------------------------ 98
xan-j.64 LAGLGLGALLDVWAGHDFPIIFYLALGGSLLFYIYSAPPLKLKQNGWIGNFALGASYIGL 240

xan-j    PWWAGQALFGTLTPDIVVLTTLYSIAGLGIAIVNDFKSIEGDRTLGLQSLPVAFGMDTAK 300
xan-j.19 PWWAGQALFGTLTPDIVVLTTLYSIAGLGIAIVNDFKSIEGDRTLGLQSLPVAFGMDTAK 300
xan-j.59 ------------------------------------------------------------ 98
xan-j.64 PWWAGQALFGTLTPDIVVLTTLYSIAGLGIAIVNDFKSIEGDRTLGLQSLPVAFGMDTAK 300

xan-j    WICVGAIDITQLSVAAYLLSTGKLYYALALVGLTIPQVILQFQYFLKDPVKYDVKYQASA 360
xan-j.19 WICVGAIDITQLSVAAYLLSTGKLYYALALVGLTIPQVILRFSTS--------------- 345
xan-j.59 ------------------------------------------------------------ 98
xan-j.64 WICVGAIDITQLSVAAYLLSTGKLYYALALVGLTIPQVILQFQYFLKDPVKYDVKYQASA 360

xan-j    QPFFVFGLLVTALATSH 377
xan-j.19 ----------------- 345
xan-j.59 ----------------- 98
                      xan-j.64 QPFFVFGLLVTALATSH 377
```

Supplementary Figure S2. Alignment of barley XanJ and the polypeptides resulting from the mutations in the three mutants *xan-j.19, xan-j.59* and *xan-j.64*. The predicted chloroplast transit peptide is underlined. Mutation *xan-j.59* results in an early stop codon. The *xan-j.64* mutation results in substitution of a serine with a phenylalanine. The *xan-j.19* mutation results in a frameshift. Non-native amino-acid residues are indicated by black boxes.

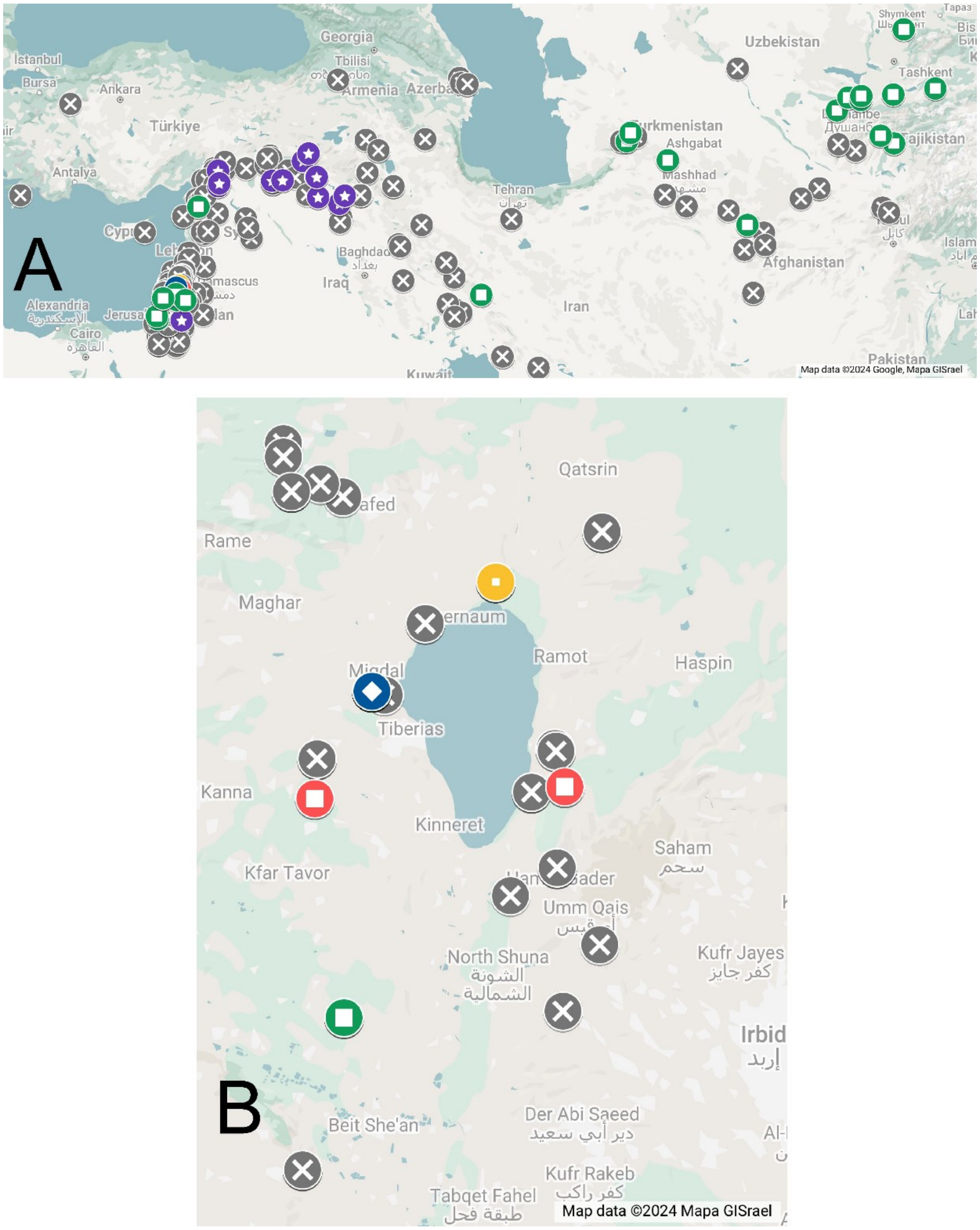

Supplementary Figure S3. Geographical distribution of barley lines with variation in the chlorophyll synthase gene *xan-j*. **A.** Lines with a Q343K substitution (purple with white star symbol) were only collected in regions on the west part of the map. The location of lines with no modifications of the polypeptide sequence or with modifications in the transit peptide have been indicated with black (with white cross) and green (with white square) dots, respectively. **B.** The wild barley lines with modifications I86T (blue with white square), V273I (yellow with white dot) and L290F (red with white square) were all collected around the Sea of Galilee.

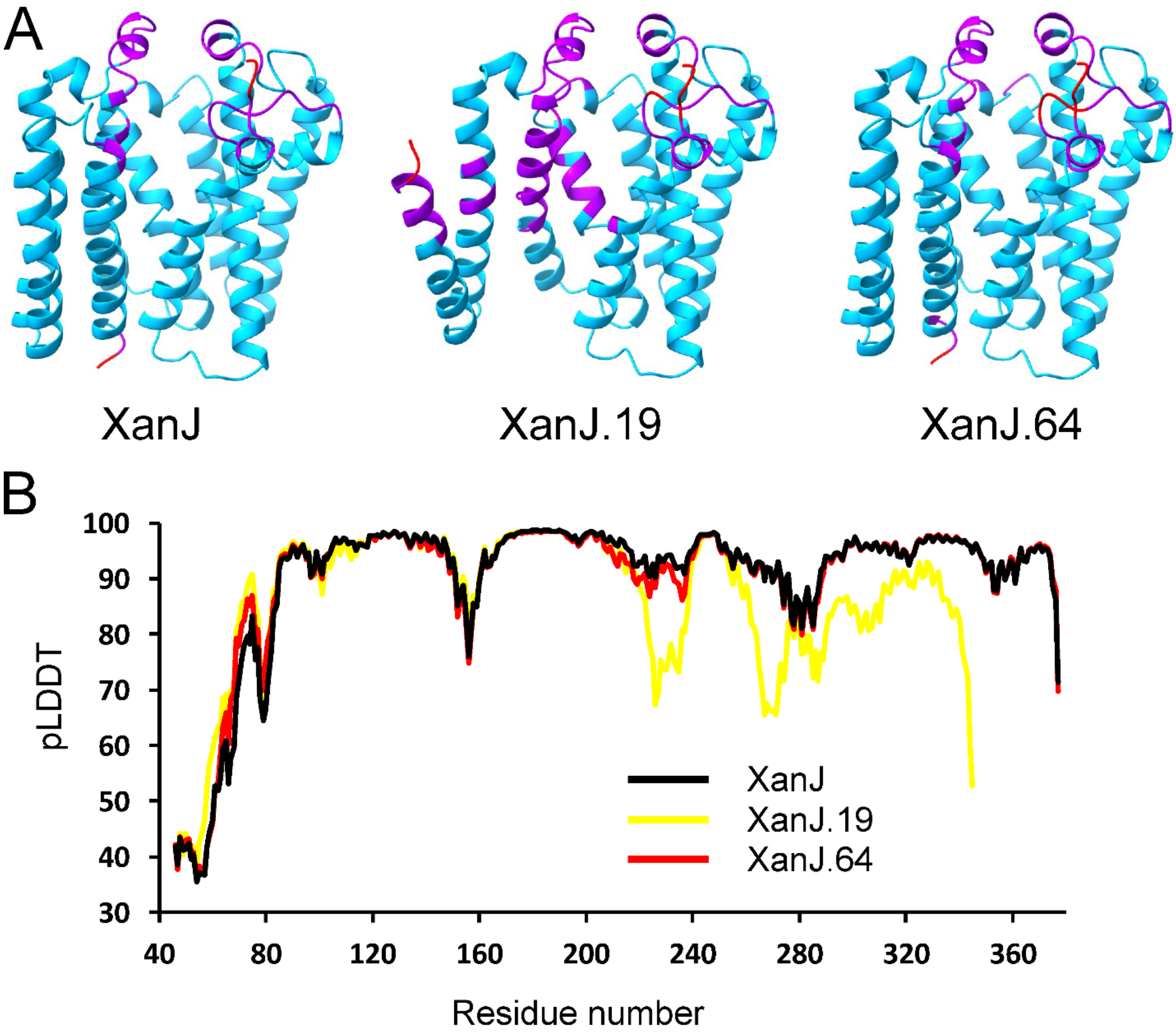

Supplementary Figure S4. Structural prediction of barley XanJ. **A.** AlphaFold generated structural models of XanJ and the proteins resulting from the mutations *xan-j.19* and *xan-j.64*. Blue parts of the structures have a pLDDT score above 90, purple between 70 and 90, red below 70. **B.** The pLDDT (predicted local distance difference test) score value for each amino-acid residues along the three polypeptides.

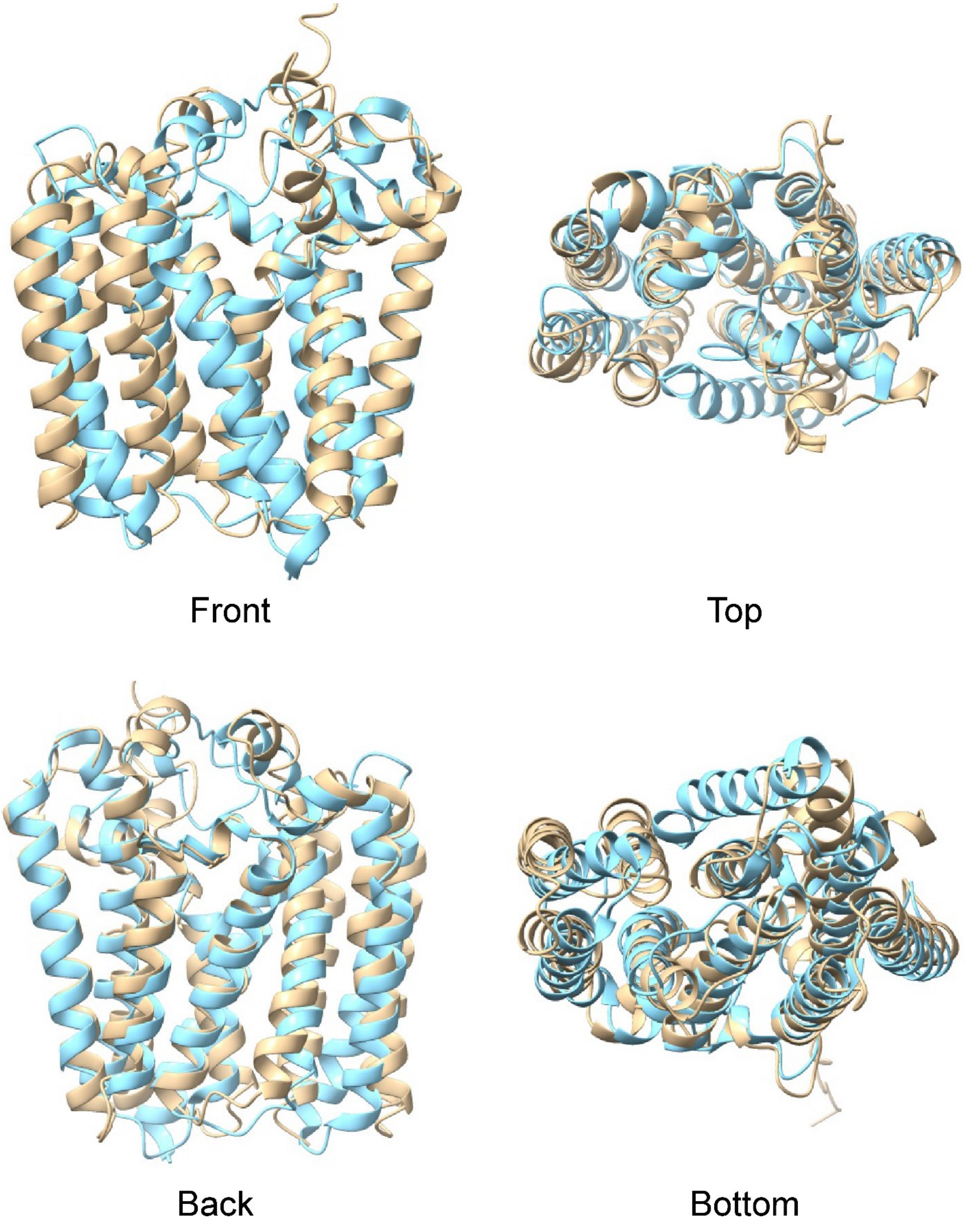

Supplementary Figure S5. Alignment of the XanJ model (brown) to the UbiA structure of *Archaeoglobus fulgidus* (blue) determined by x-ray crystallography.

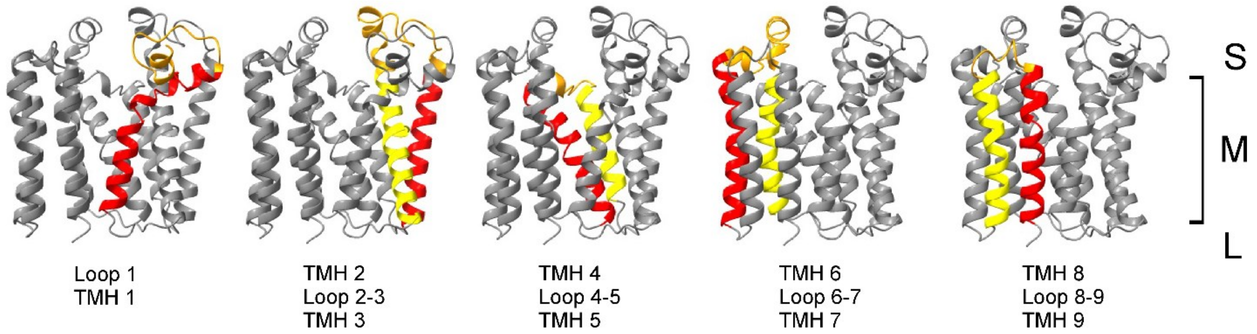

Supplementary Figure S6. AlphaFold generated structural model of barley XanJ. The five views show the consecutive order of the nine transmembrane helixes (TMH). Odd and even numbered helixes are colored red and yellow, respectively. The loops on the stroma (S) side of the membrane (M) are colored orange. L, lumen side.

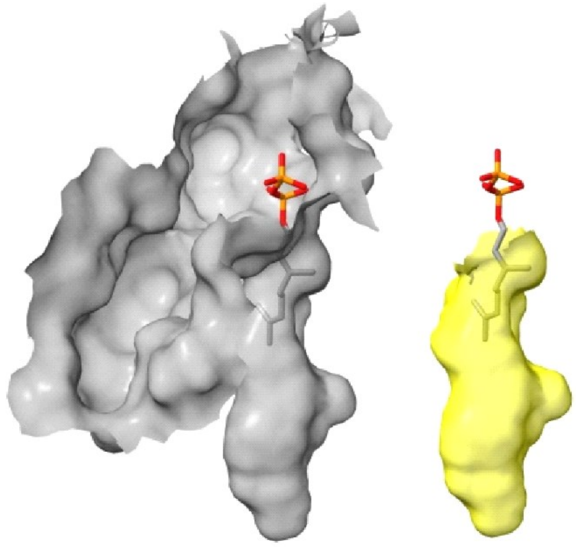

Supplementary Figure S7. The GPP in the active site of XanJ after alignment of XanJ to the GPP bound AfUbiA structure. The location of the GPP molecule suggested the binding site of GGPP/ PhyPP to an elongated cavity (yellow on the right) with the pyrophosphate in a second more globular cavity.

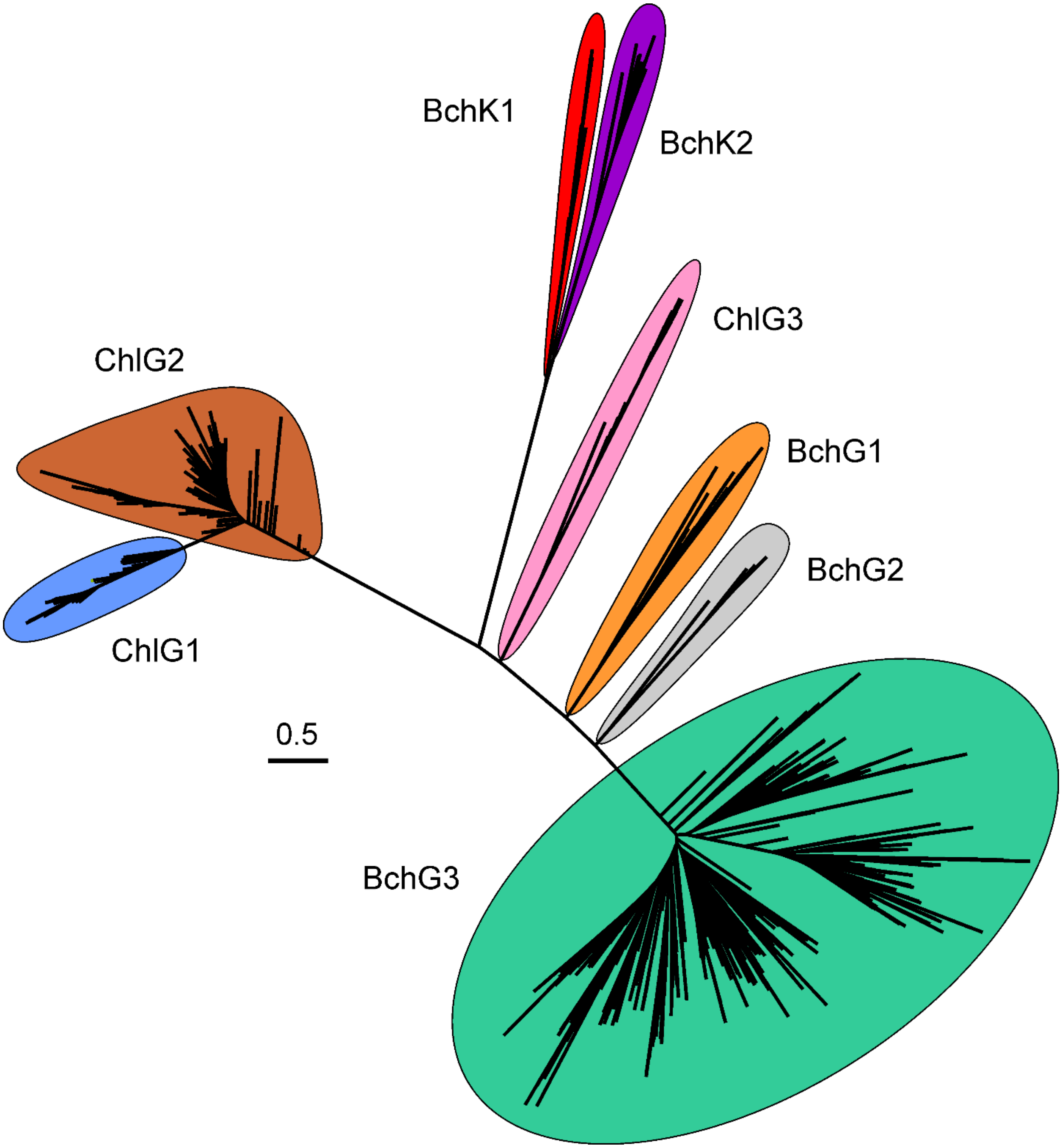

Supplementary Figure S8. Maximum likelihood phylogenetic tree based on 3749 sequences of chlorophyll synthase (ChlG), bacteriochlorophyll synthase (BchG) and bacteriochlorophyll *c* synthase (BchK). Eight groups were assigned; ChlG1, ChlG2, ChlG3, BchK1, BchK2, BchG1, BchG2, and BchG3. The scale shows Maximum likely distance.

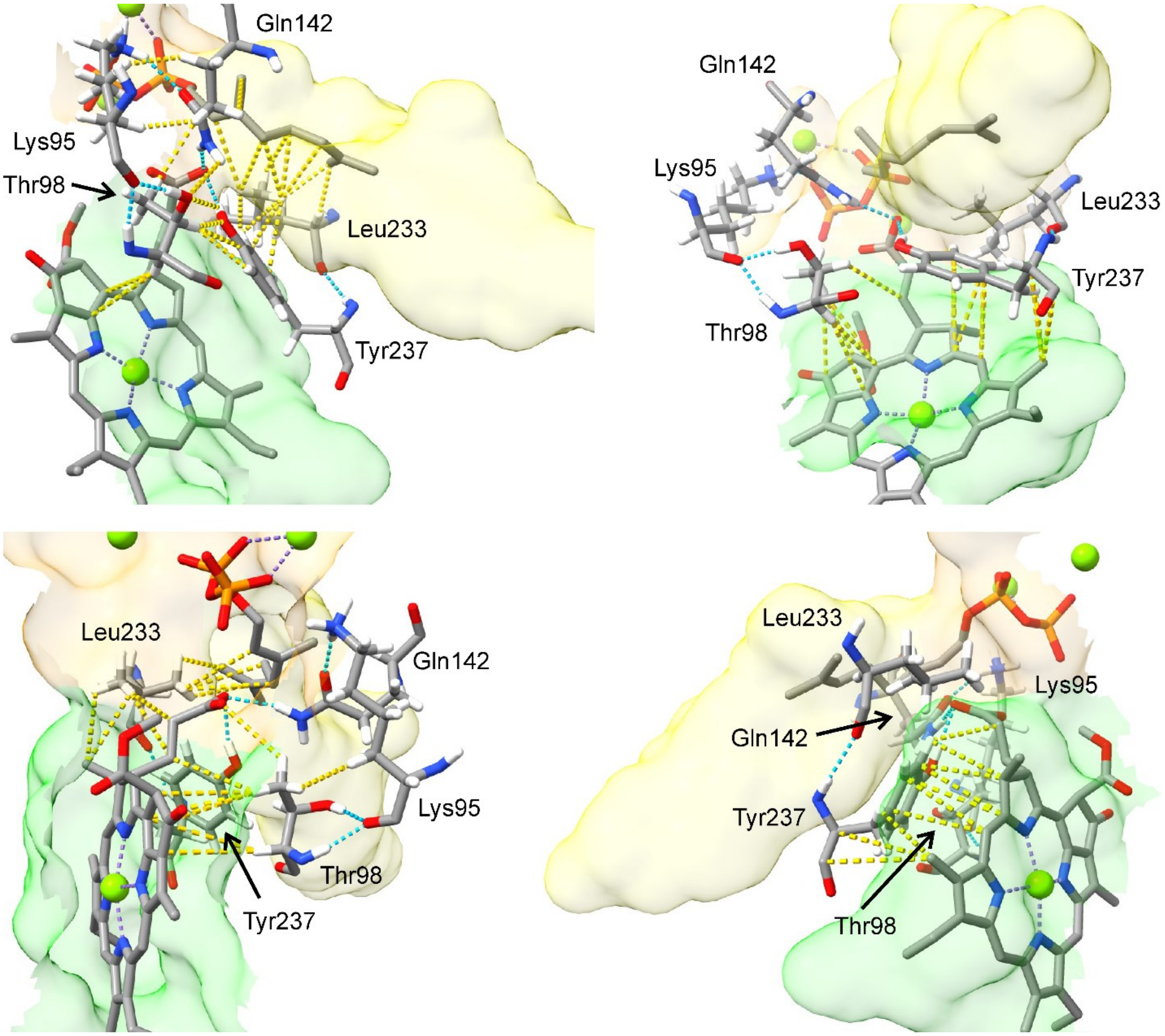

Supplementary Figure S9. Position of five key amino-acid residues in the active site of barley XanJ. Y237, Q142 and L233 are part of all three subcompartments; the PPP tunnel (yellow), the catalytic cavity (orange) and the tetrapyrrole-binding pocket (green). Y237, Q142 and T98 are suggested to interact with the carbon-17 propionate oxygens of the tetrapyrrole substrate. Chlorophyllide *a*, GPP and magnesium ions (green spheres) are also displayed in the figure. Red, oxygen; orange, phosphorus; blue, nitrogen; white, hydrogen.

SUPPLEMENTARY TABLES

Supplementary Table S1. Total number of raw 150 bp paired end reads obtained for each phenotypic bulk and the percentage of reads retained after mapping to the barley genome as well as after only retaining reads with a map quality greater than 60 (MQ60).

	xan-j.59		*xan-l.82*	
	Total number	% of raw data	Total number	% of raw data
Raw paired end reads	443,954,686	100	632,738,242	100
Mapped reads	805,748,724	90.7	1,125,755,683	89.0
Mapped reads at MQ60	491,384,209	55.3	685,391,660	54.2

Supplementary Table S2. The percentage of the barley genome covered by sequencing reads (genome coverage) and how many reads cover each of those bases (sequencing depth). The numbers for the Raw paired end reads are theoretical since they assume complete coverage.

Mutant bulk	Reads used	Genome coverage (%)	Sequence depth
xan-j.59	Raw paired end reads	100	30.7
	Mapped reads	94.5	27.6
	Mapped reads at MQ60	78.8	17.0
xan-l.82	Raw paired end reads	100	43.7
	Mapped reads	94.8	38.6
	Mapped reads at MQ60	80.8	24.2

Supplementary Table S3. Candidate genes containing homozygous mutations in the mapped chromosomal region of mutants *xan-j.59* and *xan-l.82* that alter amino acid sequence of a protein.

	Gene name	Chromo-some	Position (bp)	Confidence annotation	Functional annotation
xan-j.59	HORVU.MOREX.r2.1HG0006030	chr1H	15,716,985	HC2	SKI family transcriptional corepressor 1
	HORVU.MOREX.r2.1HG0049060	chr1H	411,433,841	HC1	Chlorophyll synthase
xan-l.82	HORVU.MOREX.r2.3HG0198710	chr3H	66,274,901	HC2	Cytosolic Fe-S cluster assembly factor NBP35
	HORVU.MOREX.r2.3HG0214090	chr3H	215,850,388	HC1	Magnesium-protoporphyrin IX monomethyl ester [oxidative] cyclase
	HORVU.MOREX.r2.3HG0221700	chr3H	330,923,492	HC2	Disease resistance protein (TIR-NBS-LRR class) family
	HORVU.MOREX.r2.3HG0224600	chr3H	362,737,081	HC1	Pentatricopeptide repeat-containing protein
	HORVU.MOREX.r2.3HG0225130	chr3H	368,518,635	HC2	Serine/threonine protein phosphatase 7 long form isogeny
	HORVU.MOREX.r2.3HG0227200	chr3H	390,534,682	HC2	D-3-phosphoglycerate dehydrogenase
	HORVU.MOREX.r2.3HG0231670	chr3H	431,514,564	HC1	Rop guanine nucleotide exchange factor
	HORVU.MOREX.r2.3HG0233580	chr3H	448,591,422	HC2	Protein phosphatase 2C

Supplementary Table S4. Natural allelic variation in *xan-j* according to a panel of 815 barley cultivars, landraces and wild barley lines (Chen et al., 2022). het – heterozygous allele.

Modification	Allele variant (ref/alt)	Bp position chr 1H	Lines
T3A	Acc/Gcc	435331513	B1K-32-02
A12AA	gcc/gccGCC	435331483	B1K-07-11 (het)
L24P	cTc/cCc	435331449	B1K-03-04, B1K-03-09 (het), FT31 (het), FT376, FT613, FT871, FT885, FT886 (het), WB-500, WBDC_007, WBDC_009, WBDC_207, WBDC_210, WBDC_212, WBDC_213, WBDC_216, WBDC_220, WBDC_225, WBDC_228, WBDC_233, WBDC_326, WBDC_336, WBDC004
P32H	cCt/cAt	435331425	B1K-29-13, WB-507
I86T	aTa/aCa	435330735	B1K-29-04
V273I	Gta/Ata	435326789	B1K-37-15
L290F	Ctt/Ttt	435326665	FT393, WB-511
Q343K	Cag/Aag	435325695	FT507, FT581, FT582 (het), FT584, FT595, FT604, FT632, FT660, FT741 (het), FT754, HID069, WB-489 (het), WB-490, WB-491, WB-497 (het), WB-498, WBDC_149, WBDC009, WBDC010

Supplementary Table S6. Primers used for PCR and RT-qPCR amplifications of *xan-j* (HORVU.MOREX.r2.1HG0049060) and RT-qPCR of *xan-f* (Olsson et al., 2004).

**Primer name**	**Sequence (5' to 3')**
ChlG_F1	ATCCATTACACGCACGGACC
ChlG_R1	CCCAATTCAACACATGCCCG
ChlG_F2	TTTGTTTTGGTGGGTTGGGC
ChlG_R2	GAACGACCATTGCAAGCCAG
ChlG_F3	TGTCTGGGGAGTGCTTTGTG
ChlG_R3	ACATGGACCCGACATTAGCA
ChlG_F4	ATCACGGCTTCTGTGCTCAT
ChlG_R4	TATCCCACGCAACTAGCACC
ChlG_F5	ACCGTCTTCGGGAGTGAGAT
ChlG_R5	TGGGGGTAACAAAAGCCTCC
ChlG_F6	AAACACAATGCACGCCTGAC
ChlG_R6	AGACAAAGTCCCTGTGCAGC
ChlG_F7	ACCGCATGCTCACAAGAAGT
ChlG_R7	ACGATCGGAAGCTCGTCATT
ChlG_F8	GCATAACTTGCCTGTCCCCA
ChlG_R8	AGCCAATGTAACTCGCACCA
ChlG_F9	GGGTGGGTCCTTGCTTTCTT
ChlG_R9	AGTGGTGTATGCCTTGGAGC
ChlG_F10	ACAACCCTAGCCCGTGATTG
ChlG_R10	GTCCCAGAGTTCTATCCCCCT
ChlG_F11	TCTCTTTTGTGCAGGTGGGC
ChlG_R11	ACTACGGCAAGCTTTGGGTG
ChlG_F12	AGGAATGGGTCGCACTTCAA
ChlG_R12	CGTCGTACTTCACAGGGTCC
ChlG_F13	GGCTTGTCAGACTGGCCTTT
ChlG_R13	ATGCATGCAGCCGCTAATAC
Xan_J_QPCR-F2	CTTGTCTGGGGAGTGCTTT
Xan_J_QPCR-R2	GTAAGACATGGACCCGACAT
Xan_F_QPCR-F2	GGGTGTGTTCAGAGACCTTT
Xan_F_QPCR-R2	TCATCTCGATTGGCTCTTCC

OLSSON, U., SIRIJOVSKI, N. & HANSSON, M. 2004. Characterization of eight barley *xantha-f* mutants deficient in magnesium chelatase. *Plant Physiol Biochem,* 42**,** 557-564.
