# Supplementary material for: A pipeline for identification of causal mutations in barley identifies Xantha-j as the chlorophyll synthase gene
Source: Plant Physiol. 2024 Apr 17;195(4):2877–90. doi: 10.1093/plphys/kiae218 (PMC11288739; doi:10.1093/plphys/kiae218)
Supplement: kiae218_Supplementary_Data [file pp.02007.2023-s04.pdf › pp.02007.2023-s04.pdf]

Plant Physiology®

A pipeline for identification of causal mutations in barley identifies xan-j as the chlorophyll synthase gene

David Stuart, Shakhira Zakhrabekova, Morten Egevang Jørgensen, Christoph Dockter, and Mats Hansson

Review Timeline:

Submission Date:	14-Dec-2023
Editorial Decision:	13-Jan-2024
Revision Received:	14-Mar-2024
Accepted:	21-Mar-2024

13-Jan-2024

Prof. Mats Hansson
Lunds Universitet
Biology
Lund 22362
Sweden

RE: A pipeline for identification of causal mutations in barley identifies xan-j as the chlorophyll synthase gene

Dear Dr. Hansson:

We have received reviews of your manuscript, which you may view at the end of this letter. On the basis of the advice received and my own evaluation, I believe that, pending revision, this article may ultimately be acceptable for publication in Plant Physiology.

When you are ready to submit your revision, click the below link and find the red arrow under your Author Tasks heading for this revision submission.

If you want to visit this version of the manuscript, you may click the link below and then click on the folder that reads "Post Decision Manuscripts".

Link Not Available

When uploading the revised version of this article, please be sure to include as complete an answer as possible to the referees' criticisms during the first round of reviews in the "response to reviewers" section.

Plant Physiology considers a revision for up to 60 days after a manuscript has been provisionally accepted; limited extension of this timeframe is at the discretion of the Monitoring Editor. A reminder notice will be sent to you 2 weeks before the deadline, at which time you may choose to contact the editorial office to request an extension; if so, please provide a justification for your request. After the expiration of this deadline, the article would need to be submitted as a resubmission and would be handled as such.

Thank you very much for giving us an opportunity to review this work. I look forward to receiving the next version.

Sincerely,

Board of Editors Plant Physiology

Want to add this revision deadline to your calendar? Click below!

--- Reviewer comments:

Reviewer #1 (Comments for the Author):

This manuscript describes the identification of causal mutations in the barley chlorophyll mutants (xan-j and xan-l) through bulked-segregant analysis combined with whole genome shotgun sequencing. In this study, the authors applied the pipeline to large-scale F2 (~1,000 seeds) mapping populations and delimited two and eight candidate mutations, respectively. Furthermore, the candidate genes were validated by the Sanger sequencing of the allelic mutants. Using cutting-edge technology such as AlphaFold2, the XanJ protein structure was characterized. Evolutionary analysis using the 3749 sequences supported the results of AlphaFold2. Overall, this manuscript is well-written and considered for publication in Plant Physiology.

Major comments

1. My question is how many genome sequence coverages are required to identify the causal mutations? Line 117, "200-300

homozygous mutants" were harvested and pooled for DNA extraction. Line 129 describes "sequencing depth of 17x and 24x". This means 5.3 Gb x 17 = 90 Gb and 5.3 Gb x 24 = 127 Gb were sequenced. However, when ~200 F2 were bulked 90 Gb and 127 Gb only covered 0.45x and 0.64x of genomes for each individual. Please clarify this point.
2. The authors are using the term "completely linked"; however, the recombination event depends on the number of chromosomes observed. I recommend modifying it to something like "did not recombine in the F2 population observed in this study".

Minor comments
1. Line 69: "recessive" > a single recessive
2. Line 88: "and" > not Italic
3. Figure 1C: please add the amino acid information.
4. Figure 2: please indicate "necrotic sections". In the legend, "A, C, D" > A, C, E
5. Figure 3B and C: The fonts on the Y-axis are too small.

Reviewer #2 (Comments for the Author):

1- The manuscript provides a very comprehensive pipeline for bulk segregant analysis coupled with genome sequencing for the identification of causal mutations in barley, with lots of methodological details in the results section. The SNP filtration steps greatly reduced the number of candidate genes/mutations, considering that the genomic regions associated with the phenotype through BSA were quite large, especially for xan-l. These filtration steps however pointed to homozygous SNPs in high-confidence mapped genes that altered the amino-acid sequence. It worked well for the two xan mutants, but other possible variants (e.g. >1bp deletions, small genomic rearrangements, variants in regulatory regions) should probably be considered when suggesting the adoption of this methodology to any mutant.

2- After cloning the gene responsible for three independent xan-j mutations, the manuscript describes an AlphaFold-derived 3D structural model of the barley chlorophyll synthase protein. The identification of the active site is reasonably supported by solved structures from related enzymes and by phylogenetic analyses to detect conserved amino-acidic positions. Based on the most recent barley genome annotations, chlorophyll synthase is a single-copy gene, and it is probably highly conserved. Still, the authors might look at natural allelic variants for chlorophyll synthase in the barley germplasm, to see if different alleles are present and if they are associated with e.g. variation in chlorophyll content or with adaptation to sites with lower/higher solar radiation.
The experiments with clomazone provide elegant hypotheses on the differences in light sensitivity of the different xan-j mutants and on the GGPP/PhyPP sensing role for chlorophyll synthase.

3- Other than the two suggestions above, I have very few comments:
- Lines 368-370: were the authors able to detect the point mutation of xan-l.82 (TGG to TGA) among the filtered SNPs?
- Line 373: from Figure 1B both telomeric regions (short and long arms) are associated with the mutation. Since the authors are providing such a detailed pipeline for BSA-seq mapping, they might discuss why for xan-j there are two peaks on the different chromosomal arms and how to interpret the allele depth difference profile in these cases. Also, it seems to me that for xan-j there is a low overlap between the BSA-50K mapping (Figure 1A) and the BSA-seq mapping, i.e. the telomeric regions associated with the phenotype by BSA-seq are not fixed for the Bonus allele in the BSA-50K mapping. Maybe two different physical maps are used?
- Line 139: what about the SNPs between Bonus and Morex (reference genome used for reads mapping) and between Quench and Morex?
- Line 151: further analyses
- Line 178: "shows" better than "obtain"

PP2023-RA-02007

Answers to comments given by reviewers

Reviewer 1

Major comments

1. My question is how many genome sequence coverages are required to identify the causal mutations? Line 117, "200-300 homozygous mutants" were harvested and pooled for DNA extraction. Line 129 describes "sequencing depth of 17x and 24x". This means 5.3 Gb x 17 = 90 Gb and 5.3 Gb x 24 = 127 Gb were sequenced. However, when ~200 F2 were bulked 90 Gb and 127 Gb only covered 0.45x and 0.64x of genomes for each individual. Please clarify this point.

ANSWER: During the planning of the experiment, we discussed internally how many mutants we should analyze and what sequence depth we should aim for. Five F2 homozygous mutants and a very low sequencing depth would certainly be enough to map the mutation to the correct chromosome since there would always be some linked markers to use. However, we also wanted to use the same dataset to identify the mutation at DNA level. There we had to consider the likelihood of getting reads covering the mutation. With a planned sequencing depth of 25-30x coverage (and obtained depth of 17x and 24x), we thought we would be on the safe side, which turned out to be correct since the two genes and their respective mutation were found. We chose 200-300 mutants since that used to be a good number when we previously had done ordinary mapping of genes. Since we did not try different numbers of mutants and sequence depths in order to optimize the process (we do not have the economic resources), we just write in the manuscript what we actually did. We are currently using our approach to identify mutations in other mutants so soon we will be able to say more.

2. The authors are using the term "completely linked"; however, the recombination event depends on the number of chromosomes observed. I recommend modifying it to something like "did not recombine in the F2 population observed in this study".

ANSWER: We changed the text to "…is completely linked to the phenotype in the analyzed dataset." The last 4 words are new.

Minor comments

1. Line 69: "recessive" > a single recessive

ANSWER: Changed as suggested.

2. Line 88: "and" > not Italic

ANSWER: Has been corrected.

3. Figure 1C: please add the amino acid information.

ANSWER: The effect of the mutations on the protein has been added to the figure text. It is already shown in detail in Supplemental Figure 2.

4. Figure 2: please indicate "necrotic sections". In the legend, "A, C, D" > A, C, E

ANSWER: Arrows have been added to indicate necrotic sections. The error has been corrected in the legend.

5. Figure 3B and C: The fonts on the Y-axis are too small.

ANSWER: Corrected as suggested.

Reviewer 2

1- The manuscript provides a very comprehensive pipeline for bulk segregant analysis coupled with genome sequencing for the identification of causal mutations in barley, with lots of methodological details in the results section. The SNP filtration steps greatly reduced the number of candidate genes/mutations, considering that the genomic regions associated with the phenotype through BSA were quite large, especially for xan-l. These filtration steps however pointed to homozygous SNPs in high-confidence mapped genes that altered the amino-acid sequence. It worked well for the two xan mutants, but other possible variants (e.g. >1bp deletions, small genomic rearrangements, variants in regulatory regions) should probably be considered when suggesting the adoption of this methodology to any mutant.
ANSWER: We used BCFtools for variant calling when we searched for candidate genes in the mapped regions. (For mapping we only used SNPs.) BCFtools is capable of identifying SNPs and also small indels. We corrected the text in order to clarify that our method is not only restricted to SNPs but also small indels. If larger genomic rearrangements are expected other variant calling programs designed for identifying structural variants can be used (for example Delly or BreakDancer). We added a sentence about this. When preparing F2-mapping populations, we intentionally choose mutants induced by chemicals over neutron and x-ray irradiation since chemically induced mutants are more likely to have "small" mutations which we believe are more likely to be identified and do not require a second round of variant calling with another tool.

2- After cloning the gene responsible for three independent xan-j mutations, the manuscript describes an AlphaFold-derived 3D structural model of the barley chlorophyll synthase protein. The identification of the active site is reasonably supported by solved structures from related enzymes and by phylogenetic analyses to detect conserved amino-acidic positions. Based on the most recent barley genome annotations, chlorophyll synthase is a single-copy gene, and it is probably highly conserved. Still, the authors might look at natural allelic variants for chlorophyll synthase in the barley germplasm, to see if different alleles are present and if they are associated with e.g. variation in chlorophyll content or with adaptation to sites with lower/higher solar radiation.
ANSWER: With help of two new coauthors, we performed an analysis of the natural allelic variation in the chlorophyll synthase gene. A new short section, a new Supplemental Table and a new Supplemental Figure have been added to the Result section.

The experiments with clomazone provide elegant hypotheses on the differences in light sensitivity of the different xan-j mutants and on the GGPP/PhyPP sensing role for chlorophyll synthase.

3- Other than the two suggestions above, I have very few comments:
- Lines 368-370: were the authors able to detect the point mutation of xan-l.82 (TGG to TGA) among the filtered SNPs?
ANSWER: We have added that we could identify the TGG to TGA mutation.

- Line 373: from Figure 1B both telomeric regions (short and long arms) are associated with the mutation. Since the authors are providing such a detailed pipeline for BSA-seq mapping, they might discuss why for xan-j there are two peaks on the different chromosomal arms and how to interpret the allele depth difference profile in these cases. Also, it seems to me that for xan-j there is a low overlap between the BSA-50K mapping (Figure 1A) and the BSA-seq

mapping, i.e. the telomeric regions associated with the phenotype by BSA-seq are not fixed for the Bonus allele in the BSA-50K mapping. Maybe two different physical maps are used?
ANSWER: The two peaks in Figure 1B could be due to several reasons. For example, a chromosomal rearrangement when comparing cultivar Bonus (mother cultivar of mutant xan-j.59) and Morex (reference genome) could result in two separate genomic regions showing linkage to the mutant phenotype, but determining the exact cause is beyond the scoop of this work. Since the xan-j mutants show monogenic inheritance, we simply started with checking both regions for candidate genes and identified the chlorophyll synthase.
Concerning the 50K-mapping in Figure 1A, such mapping is usually done with single plants and not a pool of plants like we did. Single plants are either homozygous or heterozygous for an allele. A pool can be anything between. Thus, if Figure 1B shows two peaks for xan-j.59 on chromosome 1H and a value of approximately 0.8 between the peaks, we assume that we could expect a homozygous region in Figure 1A for xan-j.59 like shown in the figure. In addition, please observe that the positioning of SNPs on the x-axis in Figure 1A is not according to the physical map (which is the case in 1B) but the SNPs in consecutive order. We added a sentence to explain this in the Figure 1A figure legend.

- Line 139: what about the SNPs between Bonus and Morex (reference genome used for reads mapping) and between Quench and Morex?
ANSWER: The Morex reference is used to map reads and during variant calling. However, for mapping, only SNPs where Bonus and Quench differ can be used. It does not matter if Morex shares an allele with Bonus or with Quench as long as Bonus and Quench differ. If Bonus and Quench share an allele which is different from Morex, that SNP can’t be used for mapping and gets removed when using the allele depth difference from the control sample to filter SNPs in the sample to be mapped.

- Line 151: further analyses
ANSWER: “Further analyzes” has been changed to “Further analyses”.

- Line 178: "shows" better than "obtain".
ANSWER: “obtain” has been changed to “show”.

21-Mar-2024
Prof. Mats Hansson
Lunds Universitet
Biology
Lund 22362
Sweden

MSID: PP2023-RA-02007R1
MS TITLE: A pipeline for identification of causal mutations in barley identifies xan-j as the chlorophyll synthase gene

Dear Dr. Mats Hansson:

I am pleased to inform you that your revised manuscript "A pipeline for identification of causal mutations in barley identifies xan-j as the chlorophyll synthase gene" has been accepted for publication in Plant Physiology. Your manuscript will be checked for consistency with journal formatting and image standards by one of our Science Editors, who will contact you shortly. They may have a number of queries and suggestions for you. Please keep in mind that they work to increase the online visibility of your article and its attraction for readers from the widest of backgrounds. You will have the opportunity to review any revisions, and I hope you will work constructively with them to get the most out of your research. You will also have the opportunity to review all changes when you receive the author proofs for your article.

Once validated for publication, your article will be sent to a Science Editor for a final review of your manuscript and source files. After your final files are uploaded, your article will appear in the next available issue and the online Preview version of your article will be posted after your signed Copyright and Checklist forms and final source files are received.

Please be sure to include the following in your manuscript file:
Harvard style citations
List of all author contributions and funding information at the beginning of manuscript after the titles and author list.
List all supplemental materials associated with the article, with titles, at the end of the ms, before the references and acknowledgments section.
Complete Figure Legends for all figures appearing in the article listed before the Literature Cited.

Thank you and congratulations on your Plant Physiology paper!

Sincerely,
Board of Editors, Plant Physiology

=====
FIRST AUTHOR PROFILES
=====

We recognize the hard work that goes into being First Author of a Plant Physiology paper by publishing a "First Author Feature" for each First Author alongside your paper.

If the first author(s) would like to submit information for a feature on Facebook and Twitter, fill out the form by clicking on the link below:

Plant Physiology First Author Feature Form: https://forms.office.com/r/ZBCGZfhm8F
If you cannot access the form through the link above, please contact Rachel at rbelsky@aspb.org.

To upload an image of yourself to accompany your profile, please put your photo in the correct journal folder at the link below. You must label your image: Author First Name Last Name Article Title.

First Author Profile Image Dropbox: https://www.dropbox.com/scl/fo/xz029p7qjr2jml2rp2kmq/h?dl=0&rlkey=6fkxcaqmxx35ap152b2osy1fz

=====

IMPORTANT REMINDER: PEER REVIEW REPORTS
=====

If you opted to publish a peer review report along with your article during the original submission process, it will be prepared by the editorial staff and publicly posted with your manuscript, inside the zip file that contains any other supplemental material. As a reminder, the peer review report is a public record of all comments from editors and reviewers, as well as your prior responses, as you received them in the decision letters for each draft of your manuscript. If you agreed to publish this report and have changed your mind, or are not sure if you selected this option, please contact the editorial office as soon as possible before signing the license agreement from our publisher.

-- Reviewer comments:
